# β-actin regulates a heterochromatin landscape essential for optimal induction of neuronal programs during direct reprograming

**DOI:** 10.1371/journal.pgen.1007846

**Published:** 2018-12-17

**Authors:** Xin Xie, Robertas Jankauskas, Aslam M. A. Mazari, Nizar Drou, Piergiorgio Percipalle

**Affiliations:** 1 Science Division, Biology Program, New York University Abu Dhabi (NYUAD), Abu Dhabi, United Arab Emirates; 2 NYU Abu Dhabi Center for Genomics and Systems Biology, Abu Dhabi, UAE; 3 Department of Molecular Biosciences, The Wenner-Gren Institute, Stockholm University, 91 Stockholm, Sweden; UNITED STATES

## Abstract

During neuronal development, β-actin serves an important role in growth cone mediated axon guidance. Consistent with this notion, *in vivo* ablation of the β-actin gene leads to abnormalities in the nervous system. However, whether β-actin is involved in the regulation of neuronal gene programs is not known. In this study, we directly reprogramed β-actin^+/+^ WT, β-actin^+/-^ HET and β-actin^-/-^ KO mouse embryonic fibroblast (MEFs) into chemically induced neurons (CiNeurons). Using RNA-seq analysis, we profiled the transcriptome changes among the CiNeurons. We discovered that induction of neuronal gene programs was impaired in KO CiNeurons in comparison to WT ones, whereas HET CiNeurons showed an intermediate levels of induction. ChIP-seq analysis of heterochromatin markers demonstrated that the impaired expression of neuronal gene programs correlated with the elevated H3K9 and H3K27 methylation levels at gene loci in β-actin deficient MEFs, which is linked to the loss of chromatin association of the BAF complex ATPase subunit Brg1. Together, our study shows that heterochromatin alteration in β-actin null MEFs impedes the induction of neuronal gene programs during direct reprograming. These findings are in line with the notion that H3K9Me3-based heterochromatin forms a major epigenetic barrier during cell fate change.

## Author summary

Although β-actin plays an important role in growth cone mediated axon guidance in neurons, the potential role of β-actin in controlling neuron differentiation remains unknown. Here, we converted β-actin^+/+^ WT, β-actin^+/-^ HET and β-actin^-/-^ KO mouse embryonic fibroblast (MEFs) into chemically induced neurons (CiNeurons) by direct reprograming. We found that the up-regulation of neuronal programs was impaired in β-actin^-/-^ CiNeurons in comparison to WT ones. β-actin^+/-^ HET CiNeurons showed an intermediate level of neuronal program expression, suggesting that β-actin dosage plays an important role during direct neuronal reprograming. Importantly, the impaired up-regulation of neuron-related genes was associated with the elevated H3K9 and H3K27 methylation levels at gene loci in KO MEFs. These epigenetic changes were accompanied by the impaired chromatin association of Brg1-containing chromatin remodeling BAF complex in β-actin null cells. Together our study demonstrates that β-actin is required for the optimal induction of neuronal gene programs during direct reprograming by presetting a favorable chromatin status.

## Introduction

In mammals, six functional actin genes expressed in a tissue-specific manner form the actin cytoskeleton, a basic physical and organizational intracellular structure that dynamically regulates cell polarity, membrane properties and cell behavior [1, 2]. Actin-containing structures are involved in neuronal development, such as growth cone dynamics, dendritic spines remodeling and neuronal precursor migration [3–5]. β- and γ-actin are the two actin isoforms expressed in mammalian neurons, but they show different localization and dynamics during neuronal development [6, 7]. γ-actin seems to be evenly present in cell body, dendrites and axons, while β-actin is confined to structures undergoing remodeling such as the growth cone [7, 8]. The transport of β-actin mRNA to the growth cone is mediated by zipcode binding protein-1 (ZBP1) [9]. However, whether the localized sorting of β-actin mRNA is essential for *in vivo* neuronal development remains controversial. First, the protein synthesis in the axon is not required for the response of growth cone to guidance cues in embryonic spinal cord [10]. Second, motor-neuron specific β-actin knock-out mice show no observable defects in axonal regeneration [11].

Nevertheless, β-actin seems to be indispensable for nervous system development. In a central nervous system specific β-actin knockout mouse, the surviving adult shows abnormalities in hippocampus and cerebellum as well as localized defects in axonal crossing of corpus callosum [12]. Another β-actin null mouse is embryonically lethal, and the neural crest cells in the embryo show arrested migration and elevated apoptosis [13]. It is noteworthy that in both mouse models, the γ- and smooth muscle α-actin isoforms are up-regulated to compensate for the lack of β-actin [12, 13]. It remains to be investigated whether the lack of β-actin affects the onset of neurogenic gene programs.

There is emerging evidence that in the cell nucleus actin plays important roles in chromatin organization, transcription regulation and cell identity control [14–16]. Nuclear actin interacts with all three RNA polymerases and its nuclear level is required for optimal transcriptional activity [16, 17]. In addition, β-actin is an integral component of several chromatin remodeling complexes, such as BAF, TIP60 and INO80 complexes in yeast, flies, and humans [18]. In line with the established roles of nuclear actin in chromatin remodeling complexes, we recently showed that in the β-actin null mouse embryonic fibroblasts, the chromatin binding of Brg1 (ATPase subunit of BAF complex) is globally impaired and the nuclear heterochromatin is re-organized [15]. Importantly, changes in global H3K9Me3 levels correlate with up- and down-regulation of certain gene programs, implying that β-actin is involved in controlling certain transcriptional programs via a chromatin-based mechanism [15].

In this study, taking advantage of the established method of direct reprograming of mouse embryonic fibroblasts (MEFs) to functional neurons [19], we investigated how β-actin deficiency may affect direct neuronal reprograming, with a focus on changes in the transcriptome. Using embryonic fibroblasts from wild type, heterozygous and knockout mice for β-actin [15], we found that compromising at least one of the β-actin alleles led to dysregulation of sets of neuron-related genes. Although all three MEFs can be directly reprogramed into chemically induced neurons (CiNeurons), we discovered that the up-regulation of neuron-specific gene programs, such as a subset of *bHLH* proneural genes, were negatively affected in β-actin^-/-^ CiNeurons in comparison to β-actin^+/+^ CiNeurons. Interestingly, β-actin^+/-^ CiNeurons displayed an intermediate expression level of neuronal gene programs, implying a β-actin dosage-dependent induction of neuronal programs. The impaired expression of neuronal gene programs in β-actin^-/-^ CiNeurons correlates with elevated H3K9Me3 level and the loss of Brg1 enrichment at multiple gene loci in β-actin^-/-^ MEFs. Several members of *Zic* and *Irx* family genes that have been implicated in early neuronal precursor specification were found to be severely down-regulated in β-actin^-/-^ MEFs, which is also linked to H3K9Me3 and Brg1 changes. Taken together, our results suggest that β-actin is required to maintain the state of heterochromatin in embryonic fibroblasts. The alteration of H3K9Me3-based heterochromatin landscape in the absence of β-actin forms an epigenetic barrier that negatively impacts on the neuronal program induction during direct reprograming.

## Results

### In embryonic fibroblasts endogenous β-actin levels affect expression of neuron-related genes

**Fig 1:**
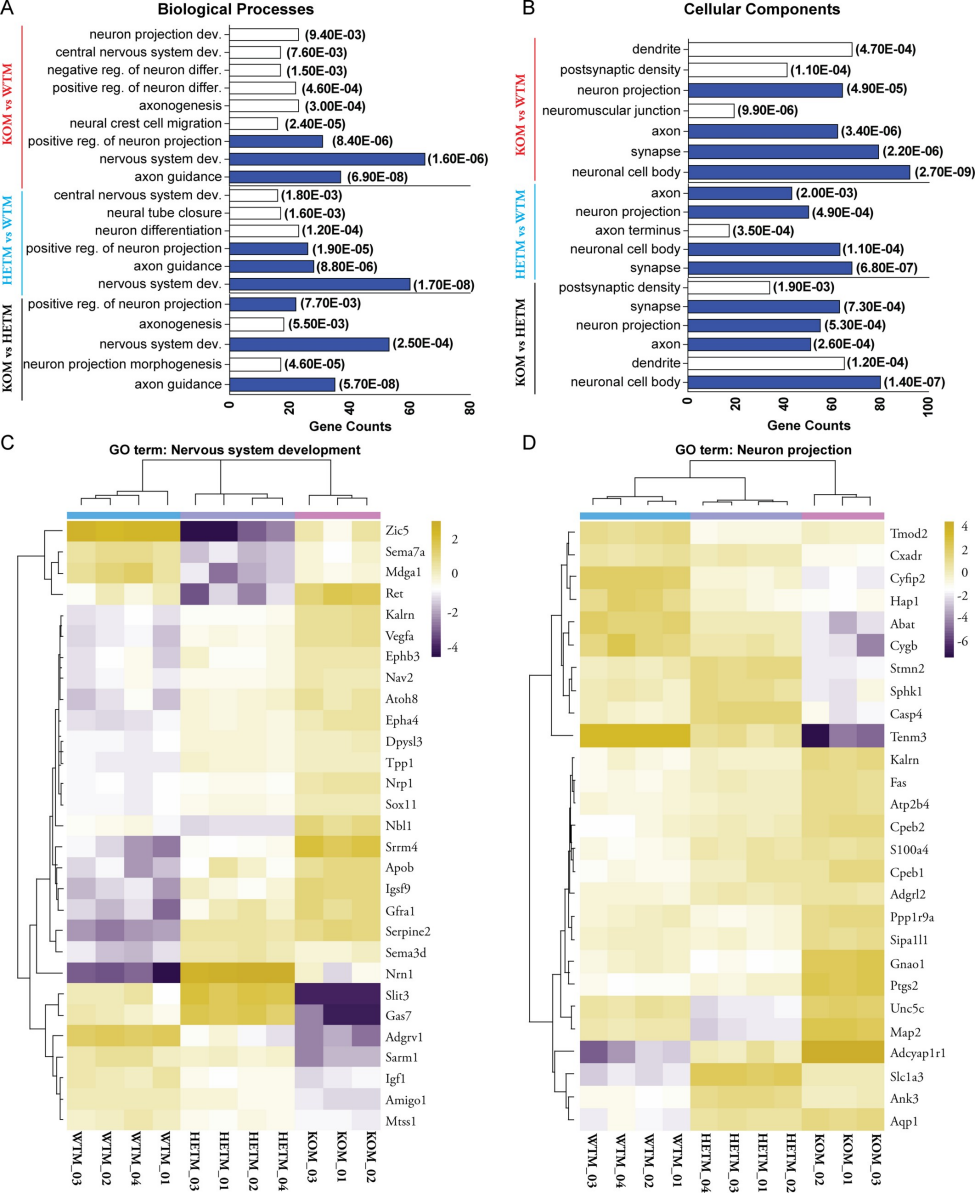
Sets of neuron-related genes are differentially expressed among β-actin ^+/+^ (WTM), β-actin ^+/-^ (HETM) and β-actin ^-/-^ (KOM) MEFs. **(A-B)** Genes differentially expressed by at least 2 fold between each group (FDR-adjusted *p* value <0.05) were subject to Gene Ontology (GO) enrichment analysis. The GO terms with gene counts ≥ 15, fold of enrichment ≥ 1.5 and *p* value <0.01 were considered to be significantly over-represented. The significantly enriched biological processes (**A**) and cellular components (**B**) that are related to neuron are shown. Numbers in parentheses show the *p* value of enrichment of each GO term. The common GO terms shared by 3 groups are labeled in blue. (**C-D)** Heatmap clustering of expression levels of genes associated with GO term: Nervous system development (**C**) and Neuron projection (**D**). Genes are selected when they are differentially expressed by at least 2 fold in WTM vs KOM comparison, and are also significantly changed in HETM vs WTM and KOM vs HETM comparisons. Clustering is based on the CV of gene expression. Scale bar: log2 CPM.

We recently reported that β-actin^-/-^ (KOM) MEFs exhibit global changes in the chromatin, extensive transcriptional reprograming and this is accompanied by alterations in their functional phenotypes [15]. Here, we studied whether the expression of genes involved in neurogenesis is also altered concomitantly with changes in the levels of intracellular β-actin. Using published RNA-seq data obtained in MEFs [15], we selected the genes differentially expressed (FDR-adjusted *p* value <0.05) by at least 2 fold between KOM vs WTM, KOM vs HETM and HETM vs WTM respectively to perform Gene Ontology (GO) enrichment analysis. We found that several neuron-related biological processes and cellular components were significantly over-represented among the differentially expressed genes (Fig 1A and 1B). For example, neural crest cell migration is affected by the differentially expressed genes between KOM vs WTM, which is consistent with the previously reported defect in neural crest cell migration in β-actin null embryo [13]. Several GO terms were shared by 3 comparison groups (Fig 1A and 1B, labeled in blue), indicating that varying levels of β-actin can impact on the same neuronal biological process or component. We also analyzed the genes that are associated with the commonly affected GO terms and found that only few genes were shared by 3 comparison groups (S1A Fig). Therefore, the varying dosages of β-actin can affect the same process in MEFs, but different sets of genes are dysregulated.

We next investigated how the expression level of genes associated with each GO term is affected by β-actin dosage. We first isolated the genes associated with nervous system development and neuron projection that are changed by at least 2-fold between KOM vs WTM. We further filtered out the genes whose expression differed significantly in both KOM vs HETM and HETM vs WTM groups (Fig 1C and 1D). Hierarchical clustering analysis showed that many of those genes showed intermediate expression level in HETM cells (Fig 1C and 1D). The same results were observed for genes involved in neuronal cell body and axon (S1B and S1C Fig). Interestingly, more than 60% of genes in each GO term showed at least 2 fold up-regulation in KOM cells when compared to WTM cells. Together, our data shows that sets of neuron-related genes are differentially affected by decreasing dosage of β-actin in MEFs. Although MEFs are not committed to neuron lineages, the differential expression of those neuron-related genes in MEFs correlating with varying β-actin levels suggests that the neurogenesis process is potentially regulated by β-actin. Therefore, we next sought to investigate whether varying levels of β-actin affect the expression of neuron gene programs by applying direct reprograming to the MEFs.

### Characterization of neuronal induction from embryonic fibroblasts of different β-actin genetic backgrounds

**Fig 2:**
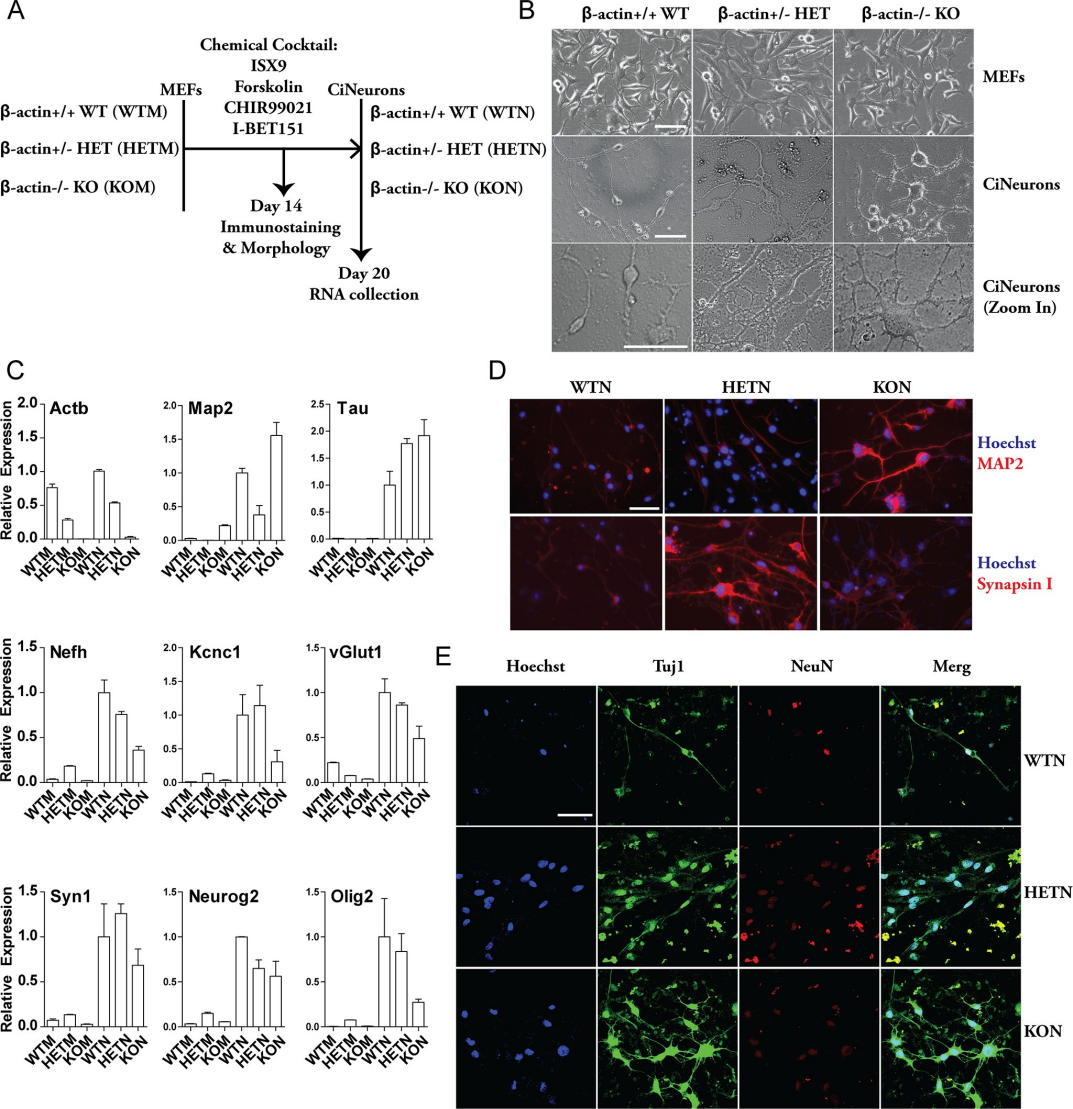
Direct reprograming of MEFs into chemically induced neurons (CiNeurons). **(A)** Schematics of experimental setup of direct reprograming of MEFs to CiNeurons by small molecule cocktail. (**B)** Cell morphology of WT, HET and KO MEFs and the corresponding CiNeurons. Scale bar: 50 μm. **(C)** qPCR quantification of relative gene expression in MEFs and CiNeurons. *Nono* housekeeping gene was used for normalization, and the expression level of WTN was set as 1 for each gene. Data are the summary of 2 independent batches of reprogramed cells. Error bar: S.E.M. **(D)** Immunofluorescence of Synapsin I and MAP2 staining in CiNeurons, Scale bar: 50 μm. **(E)** Confocal images of Tuj1 and NeuN staining in CiNeurons, Scale bar: 50 μm.

To study the possible involvement of β-actin in neuronal development, we adopted a recently established *ex vivo* protocol to directly reprogram MEFs into functional neurons by a cocktail of small molecules [19]. This protocol is based on a combination of ISX9, Forskolin, CHIR99021 and I-BET151 that disrupt the fibroblast-specific programs and activate neuron-specific programs (Fig 2A). We applied this protocol to WTM, HETM and KOM. At the end of 2-week induction when the induced cells acquired a neuronal phenotype, we captured their morphologies and immunostained the cells with neuron markers (Fig 2A). The chemically induced neurons (CiNeurons) in each β-actin genetic background are referred to as WTN, HETN and KON respectively. We found that the three CiNeurons displayed morphological differences. Typically, WTN displayed a bipolar morphology with two long extensions, while the majority of KON cells generated multiple extensions from cell body (Fig 2B). The majority of HETN also showed two major extensions. However, in contrast to WTN, we found increased number of small branches from the main extensions in HETN (Fig 2B). The cell body of WTN was significantly smaller than that of HETN and KON (Fig 2B and S2A Fig). The morphological differences can be further seen in CiNeurons stained by β-actin or α-SMA which is known to be heavily up-regulated to compensate for the loss of β-actin in KO cells [15] (S2B Fig). These observed differences in cellular morphology may be due to the altered composition of the cytoplasmic actin pool. However, our study focuses on the involvement of β-actin in the induction of neuronal gene programs at transcriptome level.

In all cell types, we revealed a general up-regulation of neuron-specific genes in the CiNeurons when compared to the MEFs counterpart. Among these genes, we found increased levels of neuron-specific microtubule associated proteins (*Map2* and *Tau*), neurofilament (*Nefh*), potassium ion channel (*Kcnc1*), glutamate transporter (*vGlut1*), Synapsin I (*Syn1*) and transcription factors (*Neurog2* and *Olig2*) (Fig 2C). The expression pattern of the β-actin (*Actb*) gene remained the same in MEFs as in CiNeurons. Noticeably, genes such as *Nefh*, *Kcnc1*, *Neurog2* and *Olig2* showed impaired up-regulation in KON in comparison to WTN. In contrast, *Map2* and *Tau* expressed at a higher level in KON (Fig 2C). Immunostaining showed that CiNeurons were positively stained with Map2, Synapsin I, Tuj1, and NeuN antibodies (Fig 2D and 2E and S2C Fig). Map2 and Tuj1 staining was much stronger in KON than WTN and HETN, suggesting a stronger microtubule assembly in KON (Fig 2D and 2E and S2C Fig). However, HETN displayed the strongest Synapsin I staining (Fig 2D and S2C Fig). Nuclei positively stained with NeuN were found in all CiNeurons (Fig 2E), however, there was a clear difference in the nuclear size. The nuclei in the WTN were found to be significantly smaller than that of HETN and KON (Fig 2D and S2D Fig), which is in line with the observation that WTM (WT MEF) nuclei are smaller than those in KOM (KO MEF) [15].

Taken altogether, the above findings indicate that the induced WTN, HETN and KON seem to exhibit general neuronal features, although their respective morphologies and expression levels of neuron-related genes are different.

### During direct reprograming, β-actin levels affect neuron-related transcriptomes

**Fig 3:**
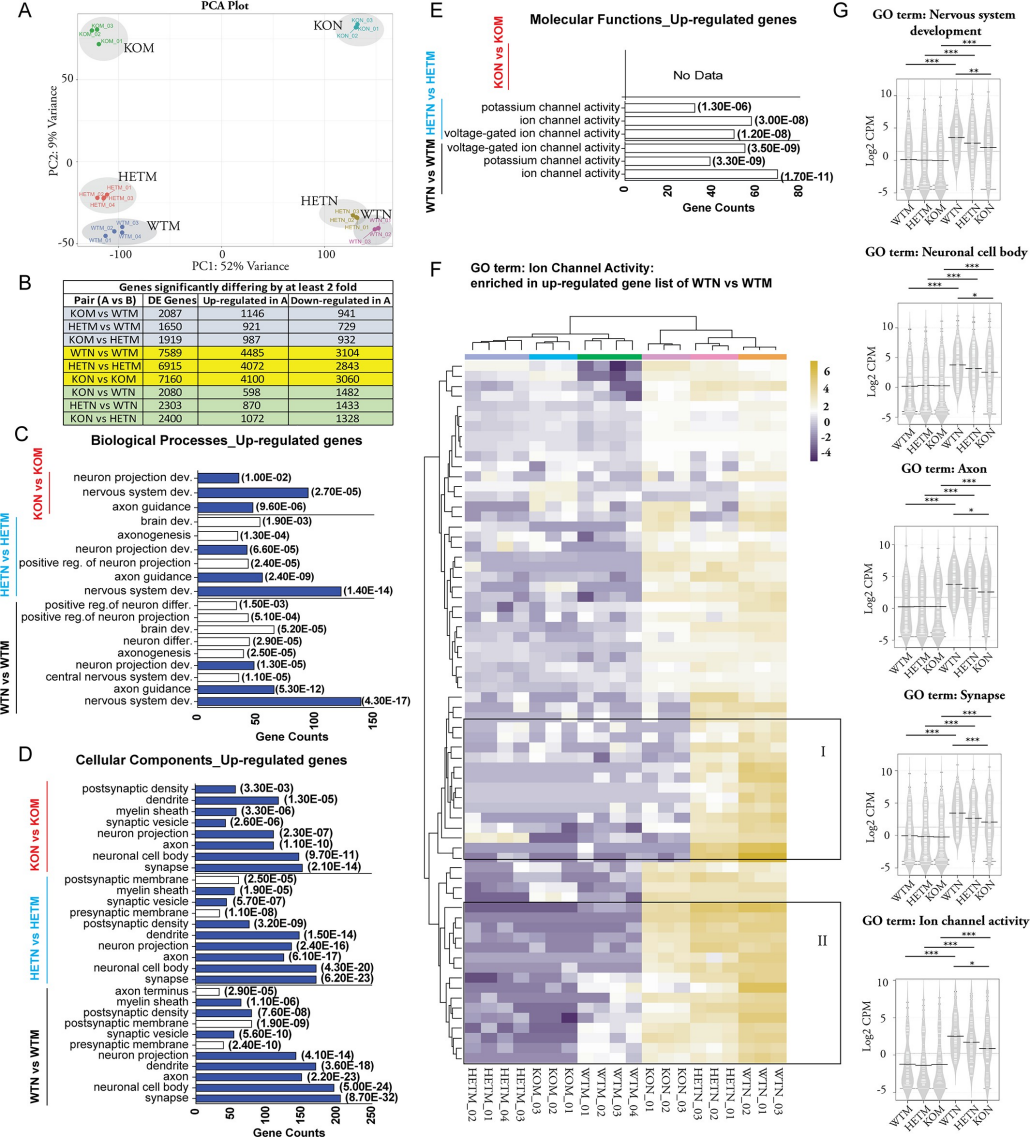
β-actin affects neuron-related transcriptome during direct reprograming in a dosage-dependent manner. **(A)** Principal component analysis of the transcriptomes of MEFs (WTM, HETM and KOM) and CiNeurons (WTN, HETN and KON). Color spheres represent individual samples. PC1 and PC2 account for 52% and 9% of the total variance, respectively. **(B)** Summary of the number of genes significantly up-regulated and down-regulated by at least 2 fold in each paired comparison. **(C-E)** Genes up-regulated in CiNeuron of each β-actin genetic background are subject to GO enrichment analysis respectively. The significantly enriched, neuron-related GO terms (gene counts of the GO term ≥ 30, fold of enrichment ≥ 1.5 and *p* value of enrichment <0.01) in Biological Processes **(C)**, Cellular Components **(D)** and Molecular Functions **(E)** are shown. **(F)** Genes that are associated with ion channel activity and are significantly up-regulated by at least 2 fold in WTN vs WTM are selected. Heatmap shows the relative expression level of those genes in all MEFs and CiNeurons. Cluster I represents genes that failed to be up-regulated in CiNeuron of KO background (KON). Cluster II represents genes that are up-regulated in KON but the expression level is lower in comparison to WTN. Scale bar: log2 CPM. **(G)** Genes up-regulated in WTN vs WTM comparison and associated with each GO term were selected. The distribution of the relative expression level (Log2 CPM) of those genes in all MEFs and CiNeurons are displayed in bean plots. Horizontal line is the mean of each sample. Statistics: One-way ANOVA with Tukey’s multiple comparison as post hoc test: * *p*<0.05; ** *P*<0.01; *** *p*<0.001.

To gain insights into the nature of CiNeurons induced from WTM, HETM and KOM, we performed transcriptional profiling by RNA-seq. Specifically, three biological replicates of RNA samples isolated from CiNeurons at Day 20 were subjected to RNA-seq analysis. We first compared the similarity of transcriptomes of all MEFs and CiNeurons. Hierarchical clustering shows that all MEF samples formed a group which clustered away from the CiNeuron samples (S3A Fig). Similarly, PCA analysis demonstrated that 52% of total variance captured by the first principle component (PC1) separated MEFs from CiNeurons, and the 9% variance by PC2 seemed to account for the difference among the 3 cell types (Fig 3A). In both MEFs and CiNeurons, the HET samples were located closer to WT samples (Fig 3A). There was a major transcriptome difference between MEFs and the CiNeurons: about 6900 to 7600 differentially expressed (DE) genes between CiNeurons and the corresponding MEFs (Fig 3B). The majority of DE genes between CiNeurons and their MEF counterparts were shared by three β-actin genetic backgrounds (S3B Fig). Noticeably, among the CiNeurons, β-actin deficiency condition led to more genes being down-regulated than those being up-regulated (Fig 3B). We also performed Gene Ontology (GO) enrichment analysis of genes commonly up-regulated or down-regulated in all CiNeurons respectively (S3C and S3D Fig). We found significant enrichment of neuron-related biological processes and cellular components, such as nervous system development, axon guidance, myelin sheath and synapse in the up-regulated genes (S3C and S3E Fig and S1 Table). In contrast, no neuron-related GO terms were significantly over-represented among down-regulated genes (S1 Table). However, the fibroblast-related GO terms such as focal adhesion and proteinaceous extracellular matrix were enriched in the down-regulated genes (S3D and S3F Fig). These results together demonstrate that direct neuronal reprograming leads to major transcriptome changes in all 3 cell types, including up-regulation of neuron-related programs and the down-regulation of fibroblast-specific programs.

Next, we sought to study the differences among the neuronal programs that are up-regulated in the three β-actin genetic backgrounds. We performed GO enrichment analysis for the up-regulated genes during reprograming in each genetic background respectively, and compared the number of GO terms identified, gene numbers associated with the GO terms and enrichment statistics. We found that apart from the commonly up-regulated biological processes and cellular components (Fig 3C and 3D, labeled in blue), cells with increasing level of β-actin tend to have more neuron-related GO terms over-represented in the up-regulated genes. We further selected the commonly identified GO terms and compared their number of gene counts and the enrichment *p* values (S4A Fig). Interestingly, with the decreasing dosage of β-actin, both the number of genes and enrichment significance associated with each GO term decreased (S4A Fig). These findings indicate that the endogenous level of β-actin affects the number of genes up-regulated in neuron-related programs during reprograming. We further selected the genes associated with nervous system development in the WTN vs WTM group, and analyzed their expression pattern in all MEFs and CiNeurons (S4B Fig). Indeed, we found that a cluster of genes showed impaired up-regulation in KON and HETN in comparison to WTN (S4B Fig, highlighted by the black frame). More strikingly, in molecular functions, the over-represented terms related to neuronal ion channel activity in WT and HET conditions failed to be found in β-actin KO condition (Fig 3E). This seems to be because a group of genes related to ion channel activity failed to be induced in KON (Fig 3F, Cluster I). It is worth noting that those genes did not seem to be differentially expressed in MEFs. There was another group of genes that were up-regulated in KON but their expression levels were lower in comparison to WTN (Fig 3F, Cluster II). These results indicate that the induction of many genes related to ion channel activity in β-actin KO condition is impaired.

To assess the expression levels of genes involved in each GO term, we selected genes in each GO term that is over-represented in WTN vs WTM group. We analyzed the distribution pattern of expression levels of those genes in all MEFs and CiNeurons (Fig 3G). Those gene programs were significantly up-regulated in all CiNeurons in comparison to MEFs. The expression levels were similar among MEFs, but the level of up-regulation in CiNeurons showed a gradual drop concomitantly with a drop in β-actin level (Fig 3G). We conclude that both GO enrichment analyses and the gene expression patterns show that the loss of β-actin negatively affects the up-regulation of neuronal programs in a dosage dependent manner.

The above comparison of CiNeurons with their MEF counterparts allows normalization for the transcriptome difference existing in MEFs. We also adopted another way to compare the transcriptomes of three CiNeurons directly. The clustering analysis of DE genes among the three CiNeurons also showed that the majority of DE genes are down-regulated in KON in comparison to WTN, whereas HETN showed an overall intermediate level of expression (S5A Fig). Seeing the massive down-regulation in KON and HETN in comparison to WTN, we further applied GO enrichment analysis to the down-regulated genes in each comparison group of CiNeurons (in comparison, we always compared the β-actin deficient CiNeuron to the β-actin sufficient one). The neuron-related processes, cellular components and molecular functions were found to be significantly over-represented in the down-regulated genes in each comparison group (S5B–S5D Fig). In contrast, no neuron-specific GO terms were over-represented in the up-regulated genes (S2 Table). Altogether these analyses further emphasize the importance of β-actin levels in the induction of neuronal gene programs.

### Neuronal and proneural gene expression is compromised in CiNeurons lacking β-actin

**Fig 4:**
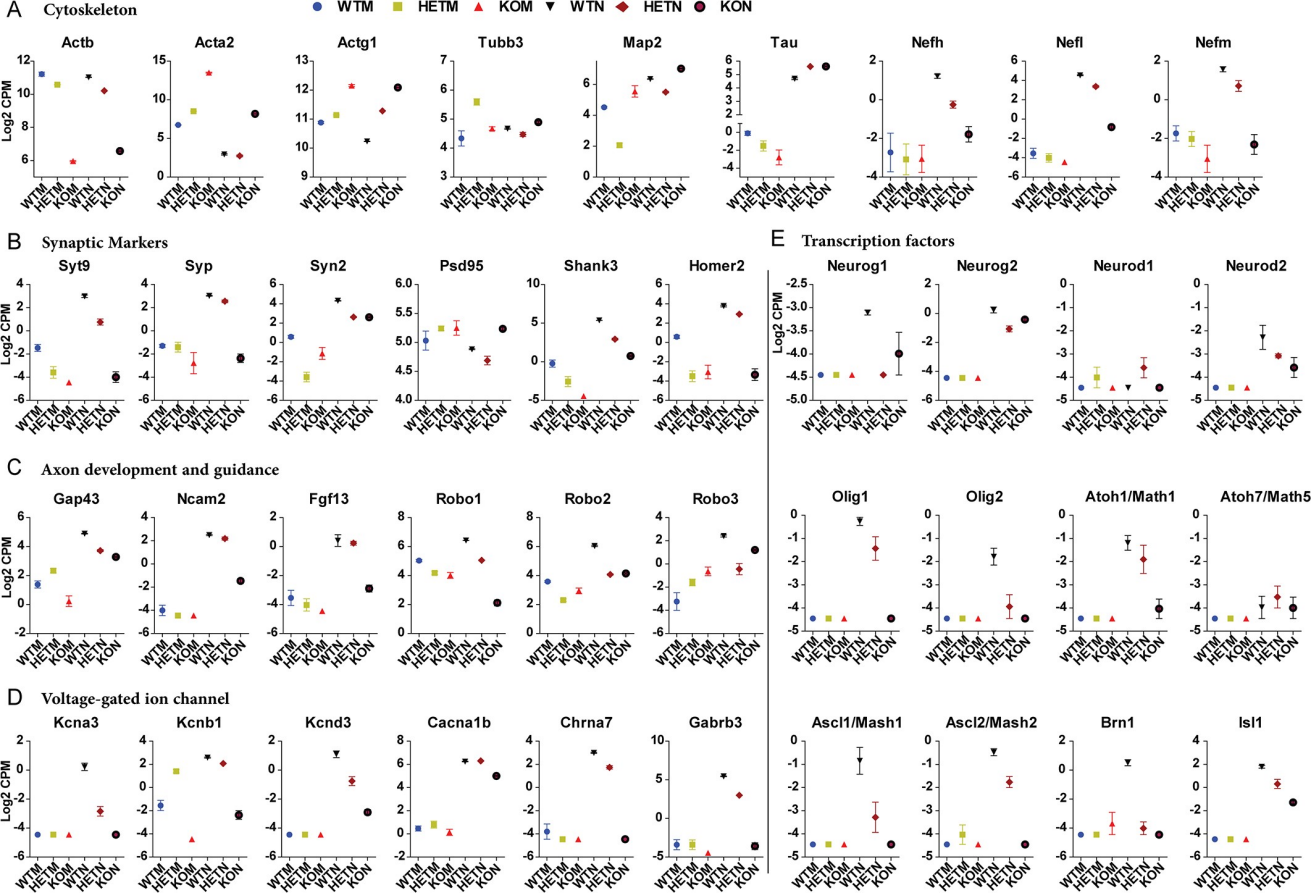
The expressions of different classes of genes in neurogenesis are affected by endogenous β-actin level. The relative expression levels of genes involved in **(A)** cytoskeleton, **(B)** synapse, **(C)** Axon development and guidance, **(D)** Voltage-gated ion channel and **(E)** Neuron-related transcription factors are shown in all MEFs and CiNeurons. The plots are the summary of at least 3 biological replicates from RNA-seq analysis. Error bar: S.E.M.

We next examined the expression patterns of different classes of neuron-related genes. For cytoskeletal genes, the relative expression pattern of *Actb* (β-actin), *Acta*2 (α-SMA) and Actg1 (γ-actin) were similar between MEFs and CiNeurons. However, the expression of *Acta*2 was reduced in all CiNeurons in comparison to the MEFs (Fig 4A). No up-regulation of *Tubb3* (Tuj1) was observed during reprograming and it was expressed at similar level in all CiNeurons, suggesting the difference of Tuj1 staining observed may be due to the different organization of tubulin. *Map2* and *Tau* were up-regulated in all CiNeurons, but were expressed at a higher level in KON in comparison to WTN (Fig 4A). For the 3 members of neurofilaments (*Nefh*, *Nefl* and *Nefm*), the up-regulation in CiNeurons showed a clear β-actin dependent pattern (Fig 4A). Together with the stronger staining of Map2 and Tuj1 observed in KON cells (Fig 2D and 2E and S2C Fig), these data suggests that in the absence of β-actin, a stronger Tuj1-based microtubule was formed in KON. However, the expression level of neurofilaments was severely impaired in KON.

Both pre-synaptic markers (*Syt9*, *Syp*, *Syn2*) and post-synaptic markers (*Shank3* and *Homer2*) were up-regulated in CiNeurons (Fig 4B). However, the post-synaptic marker *psd95* did not show up-regulation. Different genes involved in axon guidance and development were also up-regulated to different levels in CiNeurons, including the roundabout family genes (*Robo1*, *2* &*3*) involved in axon guidance, growth-associated protein 43 (*Gap43*) involved in axon growth, neural cell adhesion molecule 2 (*Ncam2*) and fibroblast growth factor 13 (*Fgf13*) (Fig 4C). Notably, there is a general tendency of impaired up-regulation of those genes in both HETN and KON in comparison to WTN. Different classes of ion channel members were also up-regulated in WTN, such as potassium voltage-gated ion channel members (*Kcna3*, *Kcnb1* and *Kcnd3*), calcium voltage-gated channel (*Cacna1b*) and ligand-gated ion channel members (*Chrna7* and *Gabrb3*) (Fig 4D). Similarly, we observed impaired up-regulation of those genes in both HETN and KON.

Proneural genes are a group of *bHLH* (basic helix-loop-helix) transcription factors that are responsible for the specification of neuron progenitor cells in ectoderm [20]. They not only function to determine neural cell fate by activating the expression of neuronal programs, but also inhibit gliogenesis [21]. We next examined different families of proneural genes, including neurogenin family (*Neurog1* and *Neruog2*), neuroD family (*Neurod1* and *Neruod2*), oligo family (*Olig1* and *Olig2*), atonal family (*Math1* and *Math5*), and achaete-scute family (Mash1 and Mash2) (Fig 4E). Those genes were generally not expressed in MEFs, and were up-regulated in WTN (except for *Neurod1* and *Mash5*). The up-regulation was either impaired or abrogated in HETN and KON, with more severe effect in KON (Fig 4E). These data show that direct reprograming can induce proneural genes but their optimal expression requires a β-actin sufficient condition. In addition, transcription factors involved in cortical neuron production (*Brn1*) and motor neuron development (*Isl1*) also showed impaired up-regulation in HETN and KON (Fig 4E). Together, our data show that the expression of both transcription factors and different neuron components are impaired in β-actin deficient conditions during direct reprograming.

### Expression of excitatory and inhibitory synaptic markers is differentially affected by β-actin

**Fig 5:**
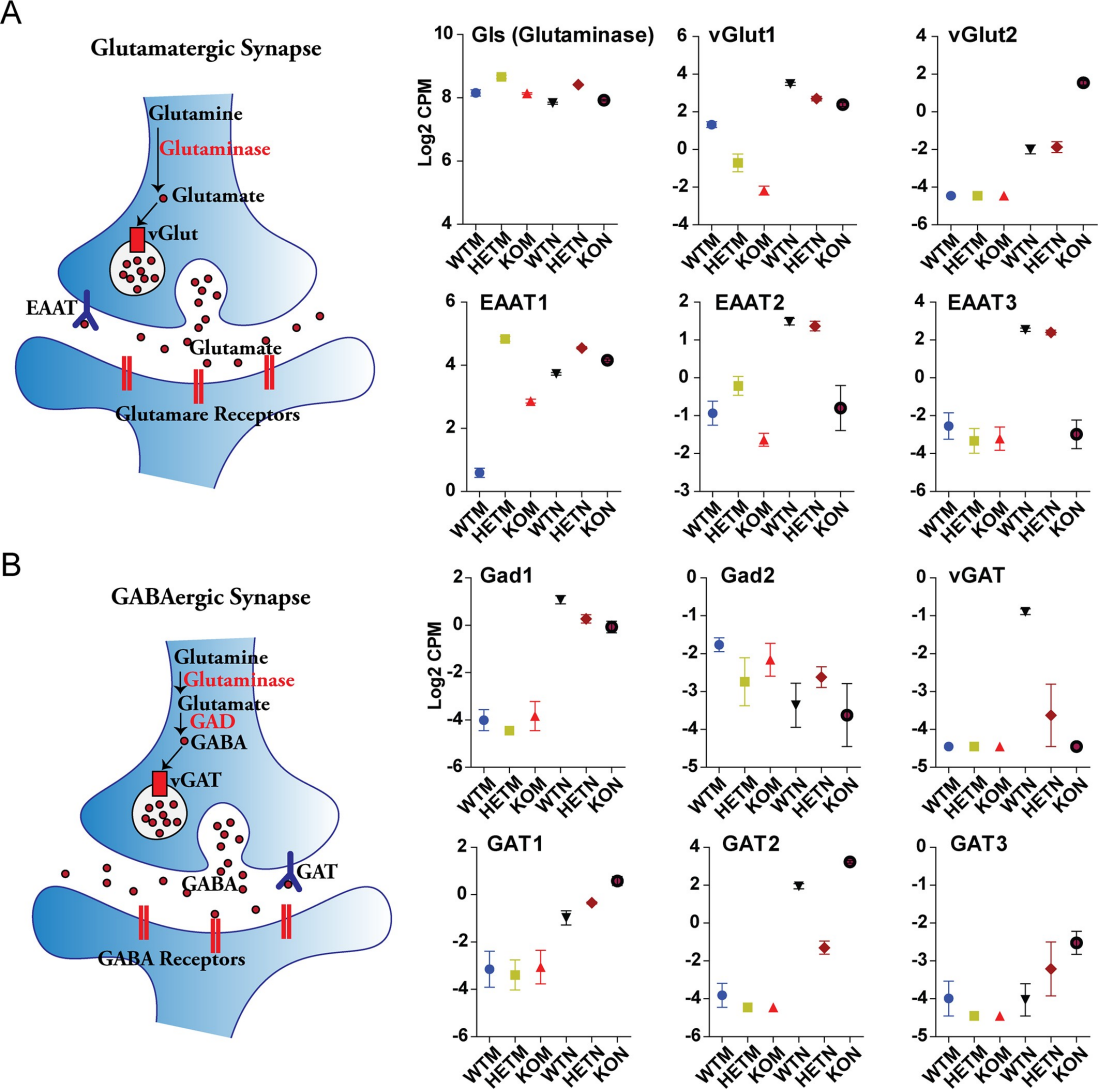
β-actin differentially affects the expression of excitatory and inhibitory neuron markers. **(A)** The schematics of key genes and processes in Glutamatergic synapse (Excitatory). The relative expressions of Glutaminase (*Gls*), *vGlut1*, *vGlut2*, *EAAT1*, *EAAT2* and *EAAT3* are shown. **(B)** The schematics of key genes and processes in GABAergic synapse (inhibitory). The relative expressions of *Gad1*, *Gad2*, *vGAT*, *GAT1*, *GAT2* and *GAT3* are shown. The plots are the summary of at least 3 biological replicates from RNA-seq analysis. Error bar: S.E.M.

Neuronal synapses can be excitatory or inhibitory based on the vesicular glutamate and GABA storage, which relies on the vesicular glutamate transporter vGlut and vesicular GABA transporter vGAT [22]. We found that the CiNeurons also up-regulated *vGlut1* and *vGlut2* genes, but *Gls* (Glutaminase) gene that was highly expressed in MEFs did not show further change in CiNeurons (Fig 5A). Nevertheless, the expression of vGlut2 was much higher in KON than WTN and HETN (see also vGlut2 staining in S2B Fig). However, the transmembrane glutamate transporter EAAT (*EAAT2* and *EAAT3*), which terminates the action of glutamate in the excitatory synapse, showed impaired up-regulation in KON in comparison to WTN and HETN (Fig 5A). In contrast, the vesicular GABA transporter (*vGAT*) up-regulated in WTN failed to be induced in KON; while the transmembrane glutamate transporter GAT (*GAT1*, *GAT2* and *GAT3*) that terminates GABA action were expressed to a higher level in KON than WTN and HETN (Fig 5B). In addition, the neuron-specific GABAergic enzyme *Gad1* was up-regulated in all CiNeurons, with the highest expression in WTN; but *Gad2* was not induced in CiNeurons (Fig 5B). Together, CiNeurons tend to differentially express GABAergic and Glutamatergic synapse markers. WTN up-regulates GABAergic synaptic marker vGAT and shows reduced expression of Glutamatergic synaptic marker vGLUT2. KON fails to up-regulate vGAT but expresses the highest level of vGLut2. Interestingly, when the CiNeurons heavily express the GABAergic or glutamatergic synaptic marker, the expression of the corresponding membrane transporter is impaired. We therefore suggest that the neurons induced from wild type embryonic fibroblasts and β-actin null embryonic fibroblasts tend to exhibit inhibitory and excitatory synapses, respectively.

### In fibroblasts, a β-actin-dependent chromatin state correlates with the expression of *Zic* and *Irx* genes

**Fig 6:**
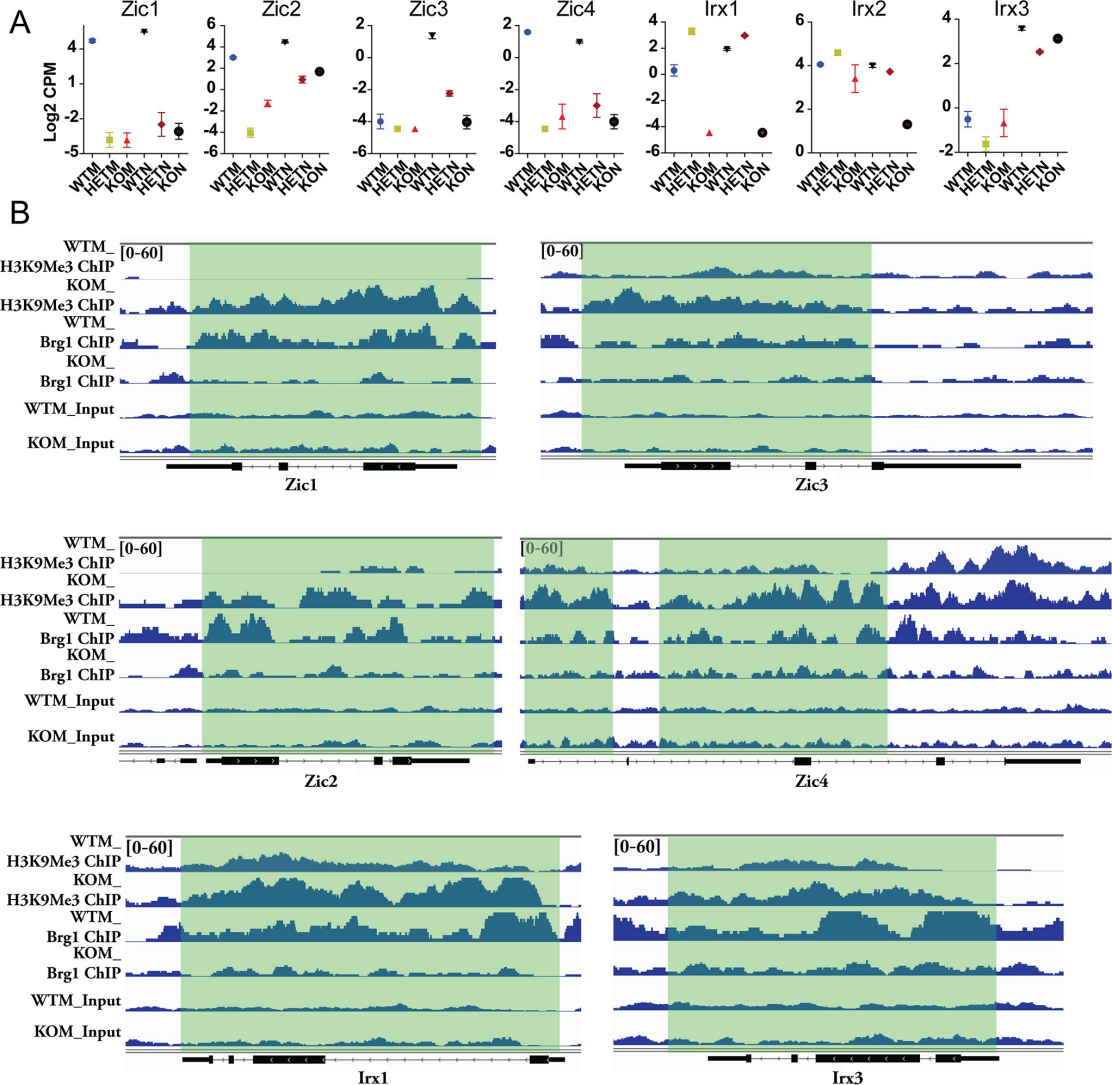
β-actin is required for the regulation of chromatin status and the expression of *Zic* and *Irx* genes in MEFs or CiNeurons. **(A)** The relative expression of *Zic* amd *Irx* genes in MEFs and CiNeurons. Data are summary of at least 3 biological replicates from RNA-seq analysis. Error bar: S.E.M. **(B)** H3K9Me3 and Brg1 ChIP-seq analysis in WTM and KOM cells at *Zic* and *Irx* loci. Normalized signal at *Zic1*; *Zic2*; *Zic3*; *Zic4*; *Irx1*; and *Irx3* loci were shown. The y-axis data range represents RPKM (Reads Per Kilobase of sequence range per Million mapped reads) per bin. The y-axis of tracks in the same image were set as the same range. Gene body position (exon: box, intron: line) are shown below the tracks. Regions of each gene loci with elevated H3K9Me3 level and impaired Brg1 binding in KOM cells are highlighted.

*Zic* (Zinc finger protein of the cerebellum) and *Irx* (Iroquois homeobox) family genes are evolutionarily conserved transcription factors involved in the earliest neural development such as neural precursor specification in embryonic neuroectoderm (NE), neural tube formation and cerebellar patterning [23, 24]. *Zic* and *Irx* genes confer NE precursors the competence to respond to neural inducing signals and promote the onset of *bHLH* proneural gene expression [25, 26]. Seeing a dramatic difference in the induction of *bHLH* genes between β-actin deficient and β-actin sufficient cells (Fig 4E), we next examined the expression of Zic and Irx genes in MEFs and CiNeurons. Surprisingly, *Zic1*, *Zic2*, *Zic4* that were highly expressed in WTM were severely down-regulated in HETM and KOM (Fig 6A). Compared to MEFs, *Zic1* and *Zic4* maintained the high expression levels in WTN and were barely induced in HETN and KON. *Zic2* was up-regulated in HETN and KON, but the expression levels were lower than WTN (Fig 6A). Zic3, which barely expressed in MEFs, was highly induced in WTN and showed impaired up-regulation in HETN and KON. In terms of *Irx* genes, *Irx1* was expressed at a much higher level in WTM and HETM in comparison to KOM, and maintained the same pattern in CiNeurons (Fig 6A). *Irx2* was expressed at a comparable level in all MEFs and showed reduced expression in KON. *Irx3* was up-regulated in all CiNeurons, with the highest expression in WTN (Fig 6A). Taken together, the above data reveal that several members of *Zic* and *Irx* genes are severely down-regulated in MEFs or show impaired up-regulation in CiNeurons when β-actin is deficient. This may negatively impact on the expression of *bHLH* proneural genes during direct reprograming since *Zic* and *Irx* genes are involved in the onset of neural fate by promoting *bHLH* expression [26].

Our recent study showed that in the absence of β-actin, the globally impaired chromatin binding of Brg1 is concomitant with an altered H3K9Me3 heterochromatin organization, which controls the up- and down-regulation of sets of genes [15]. We wondered whether the Brg1 binding and chromatin changes contribute to the observed changes of *Zic* and *Irx* genes. In line with this notion, we found a correlation between Brg1 binding, H3K9Me3 (constitutive heterochromatin marker) level and the expression level at *Zic* and *Irx* loci (Fig 6B). For Zic*1*, *2*, *3*, *4* and Irx *1*, *3* genes that showed either down-regulation in KOM or impaired up-regulation in KON, we observed that the Brg1 binding occurring at gene promoter or body in WTM was largely reduced in KOM (Fig 6B). In contrast, the H3K9Me3 level at the gene promoter or along the gene body was higher in KOM than that of WTM (Fig 6B). These data imply that the state of heterochromatin contributes to the regulation of *Zic* and *Irx* genes in mouse embryonic fibroblasts or during direct reprograming. We next expanded our analysis to study whether the heterochromatin change in MEFs contributes to an overall impaired neuronal program in the β-actin deficient condition.

### The impaired neuronal program during direct reprograming in β-actin null condition is linked to the loss of Brg1 binding and elevated H3K9Me3 at gene loci

**Fig 7:**
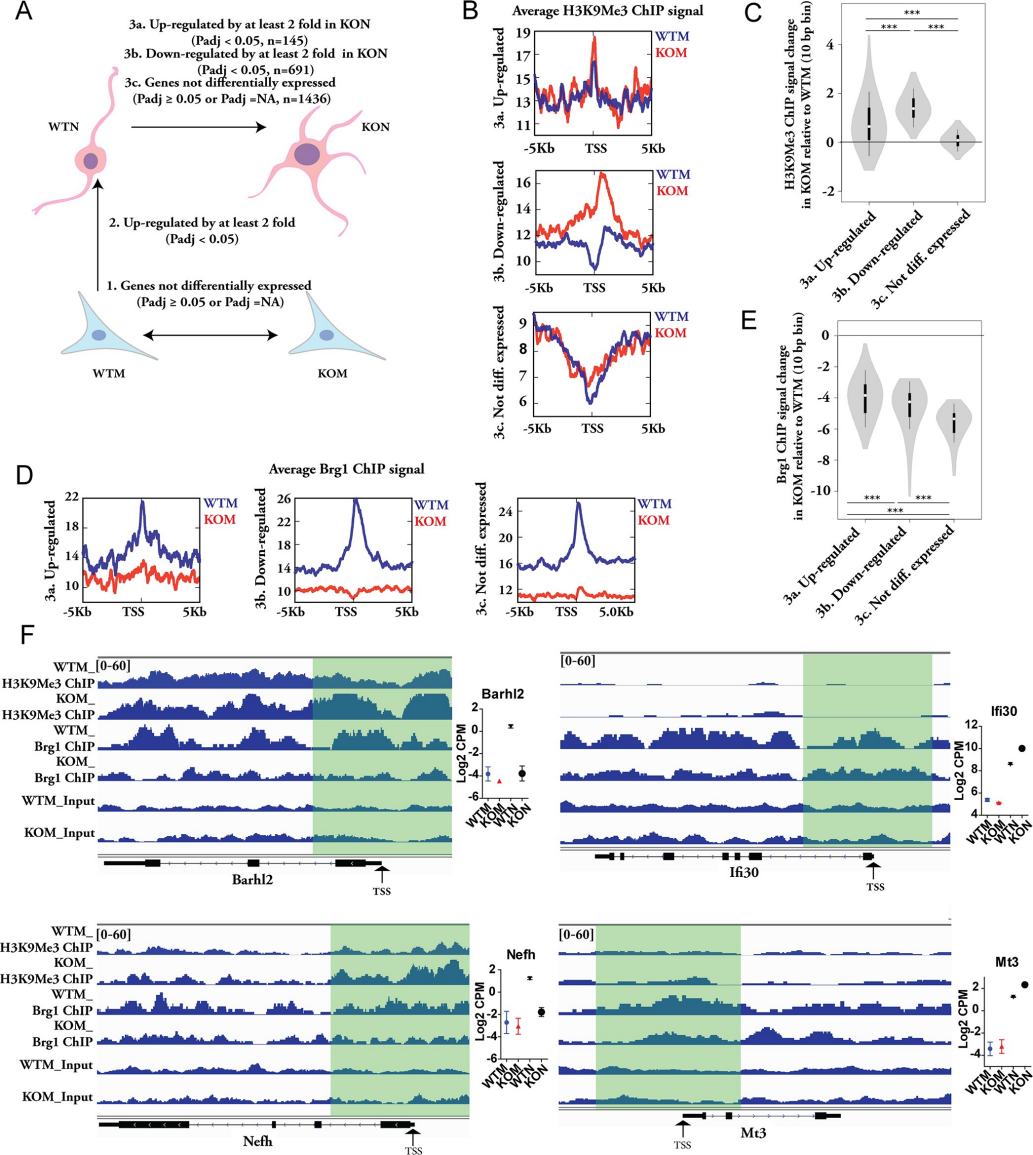
β-actin dependent Brg1 chromatin binding and H3K9Me3 changes in MEFs regulate the expression of neurogenic programs in direct reprograming. **(A)** Schematics of the selection of genes involved in neurogenesis of direct reprograming. 1. The genes differentially expressed WTM and KOM were removed. 2. After the filter 1, the remaining genes that are up-regulated by at least 2 fold in β-actin ^+/+^ WT background during direct reprograming (up-regulated in WTN in comparison to WTM) were further selected. 3. Followed by the filter 2, the genes were further filtered as follows: genes up-regulated in KON in comparison to WTN (3a), genes down-regulated in KON (3b) or genes that are not differentially expressed (3c). The final lists of filtered gene in 3a, 3b and 3c were selected respectively for the downstream analysis of H3K9Me3 and Brg1 binding profiles. **(B)** Average H3K9Me3 Chip-seq signal within ± 5kb of TSS of genes in gene lists of 3a, 3b, and 3c between WTM and KOM. **(C)** The difference of H3K9Me3 Chip-seq signal in each10 bp bin within ± 5kb of TSS between KOM and WTM was calculated. Violin diagram shows the distribution of the relative ChIP signal difference (RPKM) at each 10 bp bin between KOM and WTM. One-way ANOVA with Tukey’s post hoc test: *** p<0.001. **(D-E)** Average Brg1 Chip-seq signal within ± 5kb of TSS of genes WTM and KOM **(D)**, and the Violin diagram of the distribution of the relative Brg1 ChIP signal difference at each 10 bp bin between KOM and WTM **(E)**. **(F)** H3K9Me3 and Brg1 ChIP-seq signal in MEFs at down-regulated loci (*Barhl2* and *Nefh*) and up-regulated loci (*Ifi30* and *Mt3*) of the KON cells in comparison to WTN. The y-axis of tracks in the same image were set as the same range. Gene body position (exon: box, intron: line) are shown below the tracks. Regions of transcription start site (TSS) are highlighted. The relative gene expression plots are the summary of at least 3 biological replicates from RNA-seq analysis.

To focus on the genes involved in neurogenesis during direct reprograming, we first selected the genes that are not differentially expressed between WTM and KOM (Fig 7A, 1). Among those genes, we further filtered out the genes that are significantly up-regulated in WTN in comparison to WTM, which are the potential neurogenic genes up-regulated during reprograming under WT condition (Fig 7A, 2). After step 1 and step 2, the final gene lists were selected based on whether they are up-regulated, down-regulated or not differentially expressed between KON vs WTN (Figs 7A and 3A–3C). In this way, the final gene lists in 3a-3c stand for genes involved in neurogenesis during reprograming, but their differential expression between WTN and KON are not due to the altered expression in MEFs. We got 145 genes in 3a, 691 genes in 3b and 1426 genes in 3c respectively. To get more biological insights of the gene lists in 3a-3c, we performed GO enrichment analysis respectively. We found that a variety of neuron-specific GO terms in biological process, cellular component and molecular function were significantly over-represented in down-regulated gene list of 3b, but none of them was found in the up-regulated gene list of 3a (S3 Table). There are few neuron-related GO terms enriched in the non-differentially expressed genes (S3 Table). The analysis demonstrates that the down-regulated gene programs in KON vs WTN captured by the filtering process are highly neuron-related, while the up-regulated genes were not enriched with neuron-specific process or components. This again confirms an impaired induction of neuronal program in β-actin deficient condition, which is not due to the existing transcriptome differences in MEFs.

We next plotted the H3K9Me3 (constitutive heterochromatin marker) and Brg1 (ATPase subunit of BAF complex) ChIP-seq signal between WTM and KOM within ±5kb of the transcription start site (TSS) for the genes obtained in Fig 7A. For genes up-regulated in KON, the overall distribution of H3K9Me3 level was similar between WTM and KOM (Fig 7B, upper panel). However, a heightened level of H3K9Me3 was found in KOM at the TSS of the genes down-regulated, which was not seen in WTM (Fig 7B, middle panel). For the non-differentially expressed genes, both WTM and KOM cells showed similar pattern, with a gradual decrease of H3M9Me3 signal towards the TSS (Fig 7B, lower panel). We calculated the H3K9Me3 signal change in KOM relative to WTM for each 10 bp bin within ±5kb of TSS (Fig 7C). The violin plots showed that the overall level of H3K9Me3 increase in down-regulated genes was significantly higher than that of up-regulated or non-differentially expressed genes (Fig 7C). Notably, all the changes in the down-regulated genes were positive (above 0), indicating the elevated H3K9Me3 level in KOM for all the sub-regions within ±5kb of TSS. However, for up-regulated or non-differentially expressed genes, the changes can be either positive or negative, indicating the increased H3K9Me3 level for some sub-regions and the decreased H3K9Me3 for other sub-regions (Fig 7C). These analyses show that the elevated H3K9Me3 at a group of neuron-related loci in β-actin null MEF is specifically correlated with their impaired up-regulation in β-actin null CiNeurons, suggesting the increased H3K9Me3 level in the absence of β-actin as an epigenetic barrier for the induction of these neuron-related genes.

For the Brg1, there was a significant decrease of enrichment at TSS in all 3 gene lists in KOM in comparison to WTM (Fig 7D and 7E), indicating that the β-actin dependent chromatin binding of Brg1 is preferentially close to the TSS. The loss of Brg1 enrichment was more severe in down-regulated and non-differentially expressed genes than the up-regulated genes (Fig 7E). Together, our data show a severely reduced Brg1 binding and a higher level of heterochromatinization in KOM at the loci of genes down-regulated in KON vs WTN. For example, at loci such as *Barhl2* and *Nefh* (down-regulated in KON), there was a higher level of Brg1 binding at promoter or gene body in WTM, and the H3K9Me3 level was relatively lower (Fig 7F, left panel). In comparison, at loci such as *Ifi3*0 and *Mt3* (up-regulated in KON), no obvious changes of H3K9Me3 was observed between WTM and KOM (Fig 7F, right panel). More examples can be found in S6A and S6B Fig. The impaired Brg1 enrichment was not due to reduced gene expression or protein level in KO cells (S7A and S7B Fig).

We also profiled the H3K27 methylation (facultative heterochromatin marker) between WTM and KOM using an antibody against H3K27 dimethyl and trimethyl modification. H3K27 methylation was significantly elevated in KOM for all 3 lists of genes, suggesting an overall increased H3K27 methylation in the absence of β-actin (S7C Fig). The overall increase of H3K27 methylation in KOM is higher in down-regulated and non-differentially expressed genes than that of the up-regulated genes (S7C Fig, right panel). These data, together with an overall decreased Brg1 chromatin association, is consistent with a recent study showing that the BAF complex functions to evict Polycomb complex on chromatin to prevent the deposition of H3K27 methylation [27].

In summary, our findings show that in β-actin null mouse embryonic fibroblasts Brg1 binding at multiple gene loci is impaired. This correlates with changes in histone methylation, both H3K27 and H3K9, which are known to be involved in heterochromatin formation. The elevated levels of H3K9 and H3K27 methylation at gene loci in β-actin deficient fibroblast are likely to curb the up-regulation of genes observed during neuronal reprograming.

### Re-introduction of nucleus-targeted β-actin in KO cells affects induction of neuronal genes

To examine a direct involvement of nuclear β-actin in regulating neuronal gene programs, we applied retroviral transduction to constitutively express NLS-tagged β-actin in KOM (termed KNM cells, see S8A Fig) and reprogramed them to induced neurons. KOM cells expressing GFP were used as control for retroviral transduction (KGM cells in S8A Fig). Immunostaining shows the NLS-containing β-actin was highly enriched in the nucleus of the KNM cells (S8A Fig). It is worth mentioning that the level of re-introduced β-actin is much lower than the endogenous one in the wild type condition [15]. After reprograming, the CiNeurons obtained from control KGM cells (KGN) and those obtained from KNM cells (referred to as KNN) showed similar neuron-like morphology (S8B Fig). We quantified the expression of multiple neuronal marker genes among KGM, KNM, KGN and KNN cells by qPCR. All the marker genes showed significantly high up-regulation in CiNeurons in comparison to the corresponding MEFs (S8C Fig). When comparing KNN with KGN, we did not observe significant changes for expression of *Tau*, *Map2*, *Nefh*, *Neurog2* and *NeuroD1* genes (S8C Fig). However, we detected significant up-regulations in the expressions of *Kcnc1*, *Olig2* and *Gfap* genes in the CiNeurons constitutively expressing NLS-tagged β-actin in comparison to control cells (KNN vs KGN, see S8C Fig)). We, therefore, conclude that NLS-tagged β-actin appears to regulate the expression of certain neuron-related genes during direct reprograming, suggesting a nuclear function of β-actin in controlling neuronal gene expression.

## Discussion

In this study, we found that β-actin has an important role in reprograming mouse embryonic fibroblasts to neurons. The induction of neuron-related transcriptome appears to be dependent on β-actin dosage and is negatively regulated concomitantly with decreasing levels of endogenous β-actin. It is, therefore, likely that β-actin regulates induction of neurons from fibroblasts by controlling gene programs involved in neuronal development and function. Importantly, during the induction of β-actin null MEFs to neurons, transcriptional defects can be attributed to altered heterochromatin formation at multiple genomic loci in MEFs, as revealed by changes in the levels of the constitutive heterochromatin marker H3K9Me3. This suggests that β-actin is required to maintain and preset a favorable chromatin landscape in MEFs, which is necessary for the optimal induction of neuronal gene programs during direct reprograming.

We recently showed evidence that in β-actin null embryonic fibroblasts, both up- and down-regulation of genes correlate with decreased and increased H3K9Me3 levels, respectively, and this is dependent on β-actin dosage [15]. However, embryonic fibroblasts are not committed to neuron lineage, as manifested by the fact that the proneural *bHLH* genes specifying neuronal identity are not expressed [21, 28]. Therefore, to study the impact of β-actin dosage on the transcriptome during neurogenesis, we applied a direct reprograming method to wild-type (WTM), heterozygous (HETM) and β-actin null fibroblasts (KOM). Although the induced neurons (CiNeurons) from WTM, HETM and KOM cells commonly up-regulate neuron-related gene programs in comparison to their MEFs counterparts, in β-actin deficient backgrounds there is a clear impairment in the induction of these gene programs, including the *bHLH* transcription factors. For those genes down-regulated between KON and WTN, we detected a significantly elevated H3K9Me3 level close to the TSS in KO MEFs when compared to WT MEFs, which are not seen for the up-regulated and non-differentially expressed genes. Overall, our data suggest that the increased H3K9Me3-based heterochromatin level at those gene loci in β-actin deficient MEFs could negatively impact on the induction of those genes during neuronal reprograming. Several studies recently reported the critical role of H3K9Me3-based heterochromatin in impeding reprograming of cellular identity in somatic nuclear transfer and in induced pluripotent stem cells [29–31]. Our results are in line with the notion that H3K9Me3-dependent heterochromatin is an epigenetic barrier for cell fate change [32], and suggest that β-actin is a critical regulator of cell fate and identity, precisely by controlling heterochromatin.

Seeing that β-actin is an integral and conserved component of several chromatin-modeling complexes, β-actin is likely to regulate heterochromatin by controlling the activities of chromatin-remodeling complexes such as the BAF and INO80 complexes [18, 33]. Recently, the BAF complex has been shown to rapidly oppose the effect of polycomb repressive complexes by ATP-dependent eviction, leading to reduced H3K27Me3 levels and the formation of accessible chromatin [27]. In β-actin null fibroblasts, we observed an overall impaired chromatin binding of Brg1, the core ATPase subunit of the BAF complex, while H3K27 dimethyl and trimethyl levels are significantly increased. Since β-actin is known to regulate the activity and integrity of BAF complex [18, 33], our data suggest that β-actin maintains a functional BAF complex that in turn regulates the state of chromatin. We hypothesize that, in the absence of β-actin, the enhanced activity of polycomb repressive complex leads to the increased H3K27 methylation levels [27]. Although there is no clear mechanism as to how BAF directly affects H3K9Me3 modification, the Brg1 ATPase subunit can directly interact with H3K9Me3 binding protein HP1α, which may indicate a role for BAF in modulating HP1α-containing heterochromatin structure [34]. Indeed, we recently reported that in the absence of β-actin heterochromatin is reorganized and there is a general increase in H3K9Me3 levels [15]. In addition, silencing of another BAF complex subunit BRM is known to cause increased global H3K9Me3 levels in cancer cells [35]. We, therefore, propose that BAF complex may modulate both constitutive and facultative heterochromatin in an actin-dependent manner. The chromatin changes observed in our study may also partly originate from impaired activities of other actin-containing chromatin remodelers. Our present observations, however, provide novel evidence for one such scenario whereby loss of Brg1 chromatin association is clearly dependent on the presence of β-actin. Whether the same applies to other β-actin-containing chromatin remodelers remains to be investigated.

In mouse embryonic fibroblasts, we reported that a nuclear pool of actin controls the architectural features of the cell nucleus [15]. We revealed a similar scenario in the induced neurons. Analysis of the HETN and KON neurons induced from fibroblasts expressing suboptimal amounts of β-actin exhibited generally larger nuclei in HETN and KON in comparison to WTN. The enlarged nuclei in HETN and KON cells may result from the global heterochromatin reorganization, which is already observed in the corresponding fibroblasts, HETM and KOM [15]. Given the similarities of the nuclear phenotypes observed in fibroblasts and induced neurons, we suggest that the alterations in gene expression and nuclear architecture are caused by changes in the nuclear actin levels. Indeed, in epidermal stem cells the decreased level of nuclear actin under mechanical stretch leads to nuclear chromatin rearrangement and alterations in H3K9Me3 and H3K27Me3 levels, which controls lineage commitment [14]. Nuclear β-actin translocation is also involved in macrophage differentiation of HL-60 cells [36]. Depletion of nuclear actin seems to mediate the quiescence in epithelial cells [37], and the nuclear actin has been implicated in activating silenced genes in somatic cell reprograming [38]. We speculate that the pool of nuclear actin is important to optimize the plasticity of the genome facilitating the specification of cellular identity by controlling chromatin status. This may be exerted by regulating the activity of chromatin-remodeling complexes such as Brg1-containing BAF complex at multiple genomic loci, given the involvement of BAF complex in a wide spectrum of developmental, differentiation and reprograming processes, such as neurogenesis [39–42].

We also show evidence that members of *Zic* gene family and *Irx1* are heavily down-regulated in β-actin null fibroblasts, which seems also to be controlled by the loss of Brg1 binding and the elevated H3K9Me3 at their gene loci in the absence of β-actin. Therefore, β-actin dependent heterochromatin changes negatively affect the expression of multiple *Zic* and *Irx* genes. These genes have been shown to promote *bHLH* gene expression during neuronal fate determination in embryonic ectoderm [25, 26]. For example, the expression of *Zic* family genes sensitizes ectoderm to neural induction signals and promotes the expansion of neural progenitors in the forebrain [43, 44]. In *Xenopus*, *Irx* genes are expressed prior to the earliest expressed *bHLH* proneural genes and are required for the onset of neural differentiation [45, 46]. We therefore speculate that the severely reduced expression of *Zic* genes and *Irx1* may render the actin null MEFs less responsive to neuronal reprograming. The resulting different levels of *bHLH* proneural genes appears to impact on the development of specific neuronal subtypes. In the absence of β-actin, the induced neurons tend to express higher levels of *vGlut2* while the *vGAT* expression is greatly impaired. vGlut and vGAT are not mutually exclusive and are found to co-exist in axon terminals, which may function to regulate synaptic activity and prevent over-excitation by allowing the co-release of glutamate and GABA [22, 47]. There is evidence that inhibitory and excitatory subtypes of neurons can be specified by different combinations of transcription factors [48–50]. The varying levels of *vGlut2* and *vGAT* between WTN and KON may result from the differential expressions of a variety of transcription factors, such as the *bHLH* genes.

In conclusion, we describe a novel role of β-actin in the induction of neuronal programs during direct reprograming. We propose that these novel functions are to be ascribed to the nuclear pool of β-actin. In support of this view, we found that reintroducing NLS-tagged β-actin in β-actin KO cell nuclei can directly increase the expression of certain neuronal markers. At transcriptome and genomic level, our data show that the H3K9Me3-based heterochromatin alteration in β-actin null MEFs impedes the induction of neuronal gene programs during direct reprograming. These findings are consistent with the recent notion that H3K9Me3-based heterochromatin forms a major epigenetic barrier during cell fate change [32], highlighting a potential role of nuclear β-actin in cell fate determination by controlling the state of chromatin.

## Methods

### Antibodies and chemicals

Anti-Map2 (ab32454), anti-vGlut2 (ab79157), anti-Tubb3 (Tuj1) (ab18207), anti-NeuN (ab177487), anti-Synapsin I (ab8) and anti-H3K27Me2&Me3 (ab6147) antibodies are from Abcam. Antibody against β-actin (clone AC-74) (A5316), DMEM high glucose (D5671), Fetal Bovine Serum (F0804), Penicillin-Streptomycin (P0781), MEM non-essential amino acid solution (M7145), CHIR99021 (SML1046), Forskolin (F3917) and FGF-Basic (F0291) are from Sigma-Aldrich. Anti-GAPDH HRP (HRP 60004) is from Proteintech. Anti-total Actin HRP (sc-1615) is from Santa Cruz. Anti-Brg1 antibody is from Dr. Anki Ӧstlund-Farrants Lab (Department of Molecular Biosciences, University of Stockholm, Sweden). Hoechst 43222 (H1399), Maxima SYBR Green qPCR Master Mix (K0252), RevertAid First Strand cDNA Synthesis Kit (K1622), anti-mouse IgG Dylight 550 (84540), anti-rabbit IgG Dylight 550 (84541), smooth muscle α-actin (α-SMA) antibody (MA5-11547), Glutamax Supplement (35050061), Neurobasal medium (21103049), N2 supplement (17502048) and B27 supplement (17504044) are from Thermo Fisher Scientific. I-BET 151 (4650) and ISX 9 (4439) are from Tocris Biosciences. Trace Elements B (25-022-CI) and Matrigel matrix growth factor reduced (354230) are from Corning Inc.

### Cell culture

The mouse embryonic fibroblasts (MEFs) WTM, HETM and KOM (from the lab of Dr. Christophe Ampe, University of Gent, Belgium) were maintained and cultured with Dulbecco’s modified Eagle medium (DMEM) with high glucose, 10% fetal bovine serum (FBS) and 100 units/mL penicillin and 100 μg/mL streptomycin, in a humidified incubator with 5% CO_2_ at 37 ^o^C. The KGM and KNM cells with GFP and NLS-β actin re-introduced into KOM cells were generated by retroviral transduction as described in [15].

### Neuron induction by small chemical molecules

The protocol of small chemicals induced neuron was adopted from [19], with slight modification. Small chemical molecules were dissolved and diluted in DMSO and used at the following final concentrations: ISX9: 20 mM; Forskolin: 50 mM; CHIR99021: 20 mM; and I-BET151: 2 mM. In addition to the small molecules, the neuron induction medium (Neurobasal Medium) contains the following supplements: N2 (1X) and B27 (2X) supplements, GlutaMAX (1X), penicillin-streptomycin (100 μg/ml), bFGF (20 ng/ml), 100 μM cAMP, Non-essential Amino Acid (1X) and Trace element B (1X).

MEFs were seeded to Matrigel-coated plate (1:30 dilution in pre-cold PBS and coat overnight at 4 ^o^C, at a density of 200,000 cells per well in 6-well plate and 10,000 cells/well in 96 well plate. The MEFs were cultured in DMEM until confluent. When the cells are confluent, the DMEM was replaced with neuron induction medium with 4 small molecules. The induction medium was refreshed every two days for the first week and every 3 days for the remaining induction period until day 20. The dead cells were gently washed away when the old medium was changed. The immunofluorescence was performed after 14 days induction, and the total RNA was collected after 20 days induction.

### RNA-seq analysis

The RNA-seq of MEFs was performed as described in [15], with the accession number: GSE95830. For transcriptome analysis of induced neurons, biological triplicates of each cell type were prepared for RNA-seq analysis. The total RNA of chemically-induced neurons at day 20 were purified using RNeasy Mini Kit (Qiagen, 74106). RNA-sequencing libraries were constructed using TruSeq RNA Library Prep Kit v2 (Illumina, RS-122-2002) according to the manufacturer’s instruction. Briefly, at least 100 ng total RNA was mixed with magnetic Oligo-dT beads to purify the mRNA. The purified mRNA on beads were fragmented and primed. The first strand and second strand cDNA synthesis was performed using SuperScript Double-Stranded cDNA synthesis kit (Invitrogen, 11917020). The synthesized dsDNA was then purified using AMPure XP Beads (Beckman Coulter, A63881). After purification, the DNA sample was end-repaired and adenylated at 3’ end, followed by adaptor ligation. The cDNA was further amplified with index primers using the following protocol: 98 ^o^C 30 Sec; 15 cycles of: 98 ^o^C 10 Sec, 60 ^o^C 30 Sec, and 72 ^o^C 30 Sec; 72 ^o^C 5 Min. The PCR product was purified using AMPure XP Beads and the library quality and size was analyzed using 2100 Bioanalyzer (Agilent Genomics). Libraries with compatible index primers were pooled at equal amount and the deep-sequencing was performed using Illumina HiSEq 2500 sequencing platform (New York University Abu Dhabi Sequencing Center). RNA-seq data of induced neurons were deposited in GEO database: Accession number (GSE113733).

The RNA-seq data of MEFs and induced neurons were processed through the standard RNAseq analysis pipeline at NYUAD. Briefly, raw read alignment was performed using tophat2 v2.1.0, with the parameters “–no-novel-junctions” and “–G” when specifying the genome file. The reference genome and GFF annotation correspond to the Mus musculus GRCm38.p4 genome version. Following the tophat2 alignment, read counts mapped to each gene were generated using HTseq count. The differential expression analysis of the raw counts were performed based on the DESeq2 R library. The START Web-based RNA-seq analysis and visualization resources [51] was used to perform the differential expression test and visualization. FDR-adjusted *p* value after Benjamini-Hochberg correction for multiple-testing were used as the statistics to define the differential expression. Genes with FDR-adjusted *p* value less than 0.05 are considered to be significantly differentially expressed between two samples.

### Gene ontology enrichment analysis

Genes with FDR-adjusted *p* value less than 0.05 are selected for enrichment analyses when two different conditions are compared. Gene ontology (GO) enrichment and KEGG pathway analysis was performed using the Web-based DAVID bioinformatics resources 6.8 [52]. For the GO terms or KEGG pathway terms to be considered as over-represented or enriched in each gene list, the following criteria was applied: 1. ≥ 15 genes when comparing MEF conditions or ≥ 30 genes when comparing induced neurons to MEFs were found to be associated with the GO term or KEGG term in the database; 2. the test *p* value is less than 0.01 for the enrichment; 3. the fold of enrichment (observed number of genes in the term/expected number of genes in the term) is ≥ 1.5. When these three criteria were fulfilled, the GO terms related to neurogenesis, neuronal cell component or neuronal function were selected for detailed analysis.

### Chip-seq analysis

The detailed procedures of ChIP sample preparation and deep sequencing in MEFs were described in [15]. The accession number of H3K9Me3 and Brg1 ChIP-seq data is: GSE100096. The recently performed H3K27Me2&Me3 ChIP-seq data was also added to the existing dataset: GSE100096. For ChIP-seq data analysis, the raw reads were quality assessed using FastQC v0.11.5. The raw reads were then quality trimmed using Trimmomatic to trim low quality bases, systematic base calling errors, as well sequencing adapter contamination, specifically, the parameters used were “trimmomatic_adapter.fa:2:30:10 TRAILING:3 LEADING:3 SLIDINGWINDOW:4:15 MINLEN:36”. The surviving paired reads were then aligned against the mouse reference genome (GRCm38.p4) using Burrows-Wheeler Aligner BWA-MEM 9. The resulting BAM alignments were then processed through PICARD tools (http://broadinstitute.github.io/picard), to clean, sort and deduplicate (PCR and Optical duplicates). The processed alignments were then analyzed with DeepTools2 version 2.5.1. In details, first, BigWig files were generated from the deduplicated bam files using bamCoverage, with the following parameters,

–of bigwig (specifying the output format)
–bs 10 (specifying the bin size to 10bp)
-e (for extending the paired reads to the fragment size)
--ignoreDuplicates
--normalizeUsingRPKM (for RPKM normalization)
-b (Input bam file)
-o (output name)
-p 24 (using 24 CPU threads)

The resulting BigWig files were then passed to computeMatrix, which generates a matrix of scores per genomic region that is needed by the plotHeatmap tool. It takes as input the BigWig file and a region file (specified in BED format). The regions of interest focused on up/down regulated genes or genes not differentially expressed between WTN and KON cells. The filtering and selection process of the genes were illustrated in Fig 7A. The analysis was performed separately for each sets of genes. We used the UCSC Table Browser (https://genome.ucsc.edu/cgi-bin/hgTables) to convert the genes of interest into BED format for input in computeMatrix. The parameters used in computeMatrix were,

- reference-point
-b 5000 (5kb upstream of the Starting coordinates)
-a 5000 (5kb downstream of the Starting coordinates)

The resulting matrix combined (per set of genes), the WT, KO, and their respective inputs, for both Brg1 and H3K9Me3. Finally, plotHeatmap was used to generate the heatmaps and plots. The usual input/output and labeling parameters (-m matrix_file–out output_file–T plot_title) were used in this case.

### Immunofluorescence

Cells grown on Matrigel-coated glass cover slip or in 96-well plate were fixed by cold 70% ethanol for 15 mins at -20 ^o^C and then permeabilized with 0.5% Triton X-100 for 15 mins. After blocking by 1% BSA in PBS for one hour, cells were stained with primary antibodies against Tubb3 (Tuj1) (1:150), Synapsin I (1:50), Map2 (1:150), vGlut2 (1:150), NeuN (1:100), αSMA(1:50) and β-actin (1:100) for 2 h. Then cells were washed 3 times with TBST buffer, followed by the staining with corresponding dylight 550-conjugated secondary antibody (1:800) and Hoechst 43222 (1:6000) for 1 h. Stained cells were observed using Olympus FV1000 confocal microscope and EVOS cell imaging system (Thermo Fisher Scientific). Data were analyzed using Image J software.

### Quantitative real-time qPCR

Total RNA was extracted using RNeasy Mini Kit (Qiagen) according to the manufacturer’s instruction. 1 μg total RNA was reverse transcribed to cDNA using RevertAid First Strand cDNA synthesis Kit (Thermo Fisher Scientific). Diluted cDNA was subjected to quantitative real-time PCR analysis using Maxima SYBR Green qPCR Mix (Thermo Fisher Scientific) on Stratagene 3005 qPCR system (Agilent Technology). All expression levels of target genes were normalized to the expression of *Nono* reference gene. Primers for qPCR analysis were designed against the following genes: *Actb* (forward, TATCGCTGCGCTGGTCG; reverse, CCCACGATGGAGGGGAATAC), *Map2* (forward, ACCTTCCTCCATCCTCCCTC; reverse, TCCTGCTCTGCGAATTGGTT), *Tau* (forward, GAATGTCAGGTCGAAGATTGGC; reverse, TGGACTGGACGTTGCTAAGAT), *Syn1* (forward, CCAATCTGCCGAATGGGTACA; reverse, GCGTTAGACAGCGACGAGAA), *Nefh* (forward, GTTCCGAGTGAGGTTGGACC; reverse, CCGCCGGTACTCAGTTATCTC), *Psd95* (forward, TCCGGGAGGTGACCCATTC; reverse, TTTCCGGCGCATGACGTAG), *Kcnc1* (forward, TCCACCACTAATCTCCCTTTCTC; reverse, GACTCAGGGGAAAACATCCCA), *Neurog2* (forward, AATTACATCTGGGCGCTCACC; reverse, CGTGGAGTTGGAGGATGACG), *vGlut1* (forward, TTGTGGCTACCTCCACCCTAA; reverse, CAGCCGACTCCGTTCTAAGG), *NeuroD1* (forward, CCCTACTCCTACCAGTCCCC; reverse, GAGGGGTCCGTCAAAGGAAG), *Gfap* (forward, GGGGCAAAAGCACCAAAGAAG; reverse, GGGACAACTTGTATTGTGAGCC), *Nono* (forward, GCCAGAATGAAGGCTTGACTAT; reverse, TATCAGGGGGAAGATTGCCCA).

### Western blot

Total lysate of MEFs were collected in RIPA buffer. Immunoblot were conducted with anti-Brg1 antibody (1:1000), anti-total actin HRP (1:1000), anti GAPDH-HRP (1:1000) anti-β actin antibody (1:1000). Protein bands were developed with Clarity Western ECL Substrate (Bio-Rad) and imaged by a ChemiDoc MP Imaging system (Bio-Rad).

### Summary of statistics

Two tailed student’s T test was used for statistical evaluation of two different samples. For the comparison of multiple conditions, One-way Analysis of Variance (ANOVA) was applied, with Tukey’s multiple comparison as post hoc test. Difference with a *p* value less than 0.05 was considered as significant. The description of statistics was included in the figure legends of results part.

## Supporting information

S1 Fig Sets of neuron-related genes are differentially expressed among β-actin ^+/+^ (WTM), β-actin ^+/-^ (HETM) and β-actin ^-/-^ (KOM) MEFs. **(A)** Venn diagrams showing genes associated with Neuron-related GO terms that are differentially expressed by at least 2 fold in WTM vs KOM, WTM vs HETM and HETM vs KOM comparison. **(B-C)** Heatmap clustering of expression levels of genes associated with GO term: Neuronal cell body **(B)** and Axon **(C)**. Genes are selected when they are differentially expressed by at least 2 fold in WTM vs KOM comparison, and are also significantly changed in HETM vs WTM and KOM vs HETM comparisons. Scale bar: log2 CPM.
(TIF)

S2 Fig Immunofluorescence analysis of neuronal markers in CiNeurons. **(A)** Quantification of neuronal cell body size in CiNurons. Each point represents the value of a single cell. Data are pooled results of n ≥ 140 individual cells of at least 4 independent biological samples. **(B)** β-actin and α-SMA staining in WTN, HETN and KON cells. **(C)** SynapsinI, MAP2, Tuj1 and vGlut2 staining in WTN, HETN and KON cells. Scale bar: 50μm. **(D)** Quantification of the nuclear size in CiNurons. Each point represents the value of a single cell. Data are pooled results of n ≥ 140 individual cells of at least 4 independent biological samples. Statistics: One-way ANOVA with Tukey’s post hoc test. ns: no significant difference; *** p<0.001.
(TIF)

S3 Fig CiNeurons up-regulates neuron-related gene programs while down-regulates fibroblast-related gene programs. **(A)** Quantification of the similarity in the transcriptomes of transcriptomes of MEFs (WTM, HETM and KOM) and CiNeurons (WTN, HETN and KON). Euclidean distances were calculated from regularized log-transformed read counts. MEFs are clustered away from the CiNeurons. **(B)** Venn diagram shows the genes differentially expressed by at least 2 fold in each CiNeuron when compared to the MEF counterpart. The majority of DE genes are shared by three groups. **(C)** Venn diagram shows the genes up-regulated by at least 2 fold in each CiNeuron in comparison to the MEF counterpart. The commonly up-regulated genes shared by 3 genetic backgrounds were subject to GO enrichment analysis. The significantly over-represented terms related to neuron are shown (gene counts of the GO term ≥ 30, fold of enrichment ≥ 1.5 and *p* value of enrichment <0.01). **(D)** The same analysis is performed with the commonly down-regulated genes and the significantly enriched, fibroblast-related GO terms: focal adhesion and extracellular matrix are shown. **(E)** Heatmap showing the expression level of genes associated with GO term: Nervous system development that are commonly up-regulated in all CiNeurons. **(F)** Heatmap showing the expression level of genes associated with GO term: Proteinaceous extracellular matrix that are commonly down-regulated in all CiNeurons. Scale bar: Log2 CPM.
(TIF)

S4 Fig The expressions of neuron-related gene programs are affected by endogenous β-actin level. **(A)** Genes up-regulated in CiNeuron of each genetic background are subject to GO enrichment analysis respectively. The significantly enriched, neuron-related GO terms in Biological Processes Cellular Components are shown. Y-axis shows the number of gene counts in each GO term. Numbers in parentheses show the *p* value of enrichment of each GO term. **(B)** Genes that are associated with nervous system development and are significantly up-regulated in WTN vs WTM are selected. Heatmap shows the expression level of those genes in all MEFs and CiNeurons. Black frame highlights the genes that failed to be up-regulated in CiNeuron of KO background. Scale bar: log2 CPM.
(TIF)

S5 Fig Direct comparison of transcriptomes of CiNeurons of each genetic background. **(A)** Genes are selected when they are differentially expressed by at least 2 fold in WTN vs KON comparison, and are also significantly changed in HETN vs WTN and KON vs HETN comparisons. Clustering is based on the CV of gene expression. Scale bar: log2 CPM. **(B-D)** Genes down-regulated in each comparison group (less β-actin background compared to more β-actin background, e.g. KON vs WTN) are subject to GO enrichment analysis. The significantly enriched, neuron-related GO terms are shown in **(B)** Biological processes, **(C)** Cellular Components and **(D)** Molecular functions.
(TIF)

S6 Fig Example loci showing that the loss of Brg1 binding and increased H3K9Me3 in KOM correlates with the impaired up-regulation of specific genes during direct reprograming. **(A)** Examples showing H3K9Me3 and Brg1 ChIP-seq data of MEFs at gene loci that are down-regulated in KON vs WTN. The y-axis data range represents RPKM (Reads Per Kilobase of sequence range per Million mapped reads) per bin. The y-axis of tracks in the same image were set as the same range. Gene body position (exon: box, intron: line) are shown below the tracks. The transcription start site (TSS) of each gene is highlighted. The plots are the summary of relative gene expression level of at least 3 biological replicates from RNA-seq data. **(B)** Examples showing H3K9Me3 and Brg1 ChIP-seq data of MEFs at gene loci that are up-regulated in KON vs WTN.
(TIF)

S7 Fig ChIP-seq analysis reveals an overall elevated H3K27 methylation (H3K27Me2&Me3) in the KOM. (**A**) *Brg1*/*Smarca4* mRNA level in MEFs and Neurons by RNA-seq analysis. Each dot in the plot is a biological replicate. Student’s t-test, ns: no significant difference. (**B**) Western blot analysis of the Brg1 protein level in whole cell lysate of WT and KO MEFs. GAPDH is used as loading control. The Brg1 and total actin protein levels are comparable between WTM and KOM cells. (**C**) Left panel: Average H3K27Me2Me3 Chip-seq signal (RPKM) within ± 5kb of TSS of genes in gene lists of 3a, 3b, and 3c of Fig 6A between WTM and KOM. Right panel: The difference of H3K27Me2Me3 Chip-seq signal in each10 bp bin within ± 5kb of TSS between KOM and WTM was calculated. Violin diagram shows the distribution of the relative ChIP signal difference at each 10 bp bin between KOM and WTM. One-way ANOVA with Tukey’s post hoc test: *** *p*<0.001.
(TIF)

S8 Fig Re-introduction of nucleus-targeted β-actin into KOM shows tendency of rescuing the expression of certain neuron markers after neuronal reporgraming. **(A)** Immunofluorescence staining of KOM cells with re-introduced HA-β-actin-NLS (KNM cells). The HA-GFP is used as a control for retroviral transduction (KGM cells). The HA-β-actin-NLS staining is preferentially enriched in the cellular nucleus. **(B)** Morphology of the CiNeurons (KGN & KNN) induced from KGM and KNM cells. **(C)** qPCR analysis of the relative expression level of neuron markers among KGM, KNM, KGN, KNN cells. The gene expression difference between induced neurons KGN and KNN are analyzed by Student’s t-test, ns: no significant difference; * *p*<0.05. Results are pooled data of 3 independent biological samples.
(TIF)

S1 Table GO enrichment analysis of commonly up-regulated or down-regulated genes shared by CiNeurons in comparison to the MEF countpart. Genes commonly up-regulated or down-regulated in all CiNeurons when compared to the MEF counterpart are selected respectively, and are subject to GO enrichment analysis. The significantly over-represented GO terms in Biological Process, Cellular Component and Molecular function are shown, and their gene counts enrichment *p* value, and fold of enrichment. (Criteria for GO terms to be considered as significantly over-represented: Gene count at least 30, P value <0.01, Fold of enrichment at least 1.5).
(DOCX)

S2 Table GO enrichment analysis of genes up-regulated among CiNeurons. Genes up-regulated in each comparison group of CiNeurons (less β-actin background compared to more β-actin background, e.g. KON vs HETN) are subject to GO enrichment analysis. The significantly over-represented GO terms in Biological Process, Cellular Component and Molecular Function are shown. (Genes up-regulated in A (A vs B) by at least 2 fold; Criteria for GO terms to be considered as significantly over-represented: Gene count at least 30, P value <0.01, Fold of enrichment at least 1.5).
(DOCX)

S3 Table GO enrichment analysis of gene lists filtered in Fig 7A. The final lists of filter genes 3a, 3b and 3c in Fig 7A are subject to GO enrichment analysis. The significantly over-represented GO terms in Biological Process, Cellular Component and Molecular Function are shown. (Criteria for GO terms to be considered as significantly over-represented: P value <0.01, Fold of enrichment at least 1.5).
(DOCX)
