# Supplementary figures and images for: β-actin regulates a heterochromatin landscape essential for optimal induction of neuronal programs during direct reprograming
Source: PLoS Genet. 2018 Dec 17;14(12):e1007846. doi: 10.1371/journal.pgen.1007846 (PMC6312353; doi:10.1371/journal.pgen.1007846)

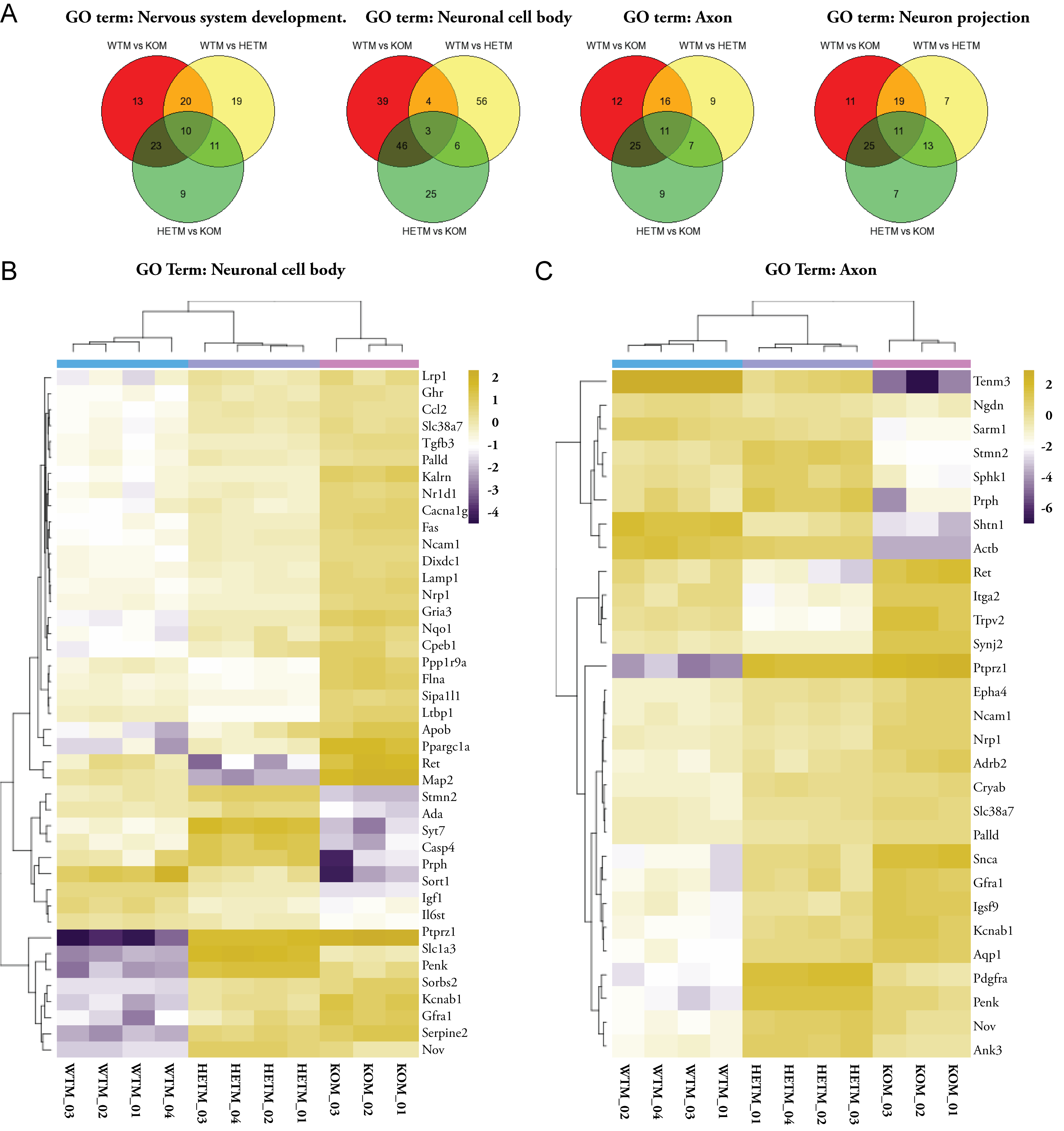

Supplement: S1 Fig — (A) Venn diagrams showing genes associated with Neuron-related GO terms that are differentially expressed by at least 2 fold in WTM vs KOM, WTM vs HETM and HETM vs KOM comparison. (B-C) Heatmap clustering of expression levels of genes associated with GO term: Neuronal cell body (B) and Axon (C). Genes are selected when they are differentially expressed by at least 2 fold in WTM vs KOM comparison, and are also significantly changed in HETM vs WTM and KOM vs HETM comparisons. Scale bar: log2 CPM. (TIF) [file pgen.1007846.s001.tif]

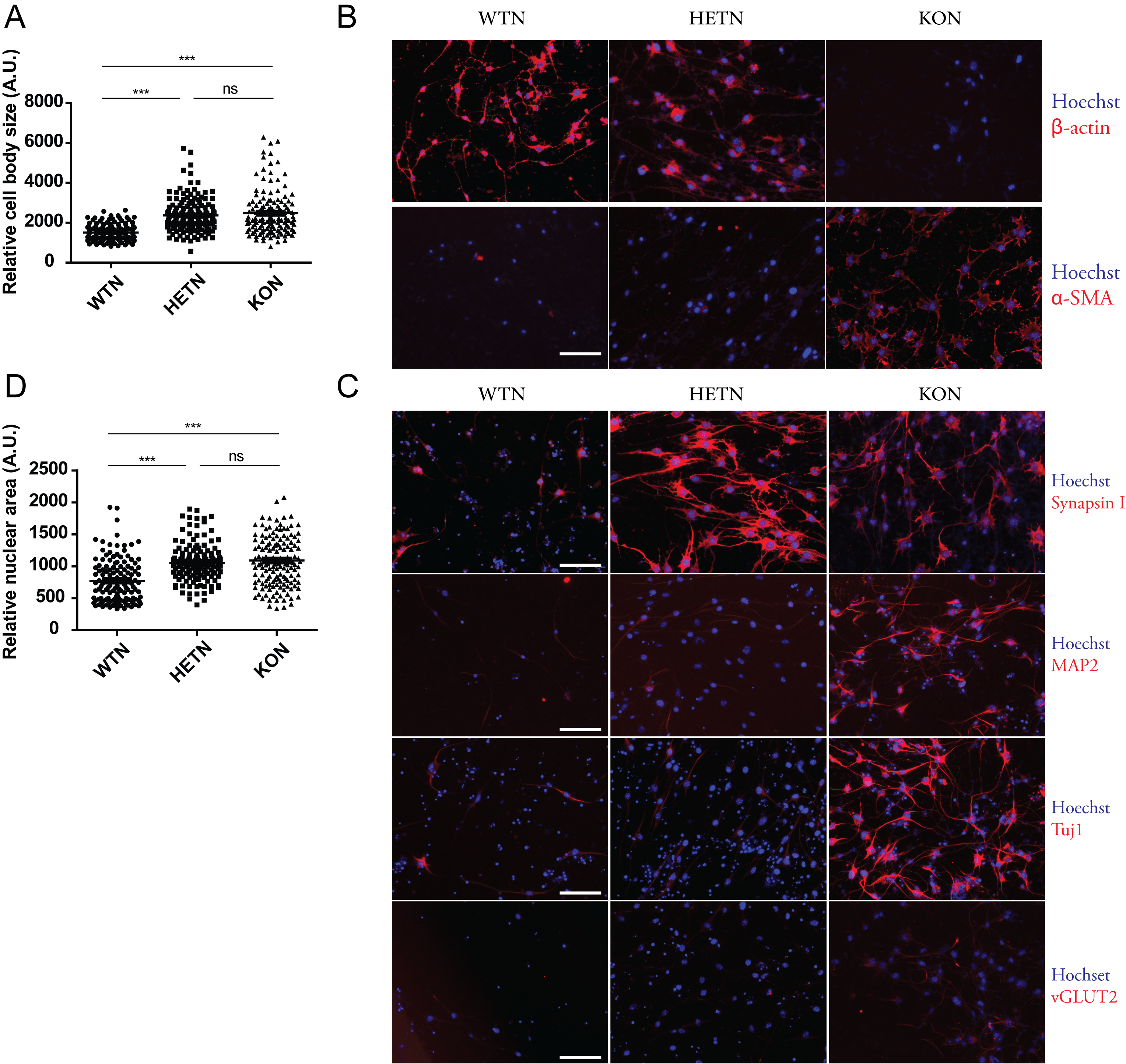

Supplement: S2 Fig — (A) Quantification of neuronal cell body size in CiNurons. Each point represents the value of a single cell. Data are pooled results of n ≥ 140 individual cells of at least 4 independent biological samples. (B) β-actin and α-SMA staining in WTN, HETN and KON cells. (C) SynapsinI, MAP2, Tuj1 and vGlut2 staining in WTN, HETN and KON cells. Scale bar: 50μm. (D) Quantification of the nuclear size in CiNurons. Each point represents the value of a single cell. Data are pooled results of n ≥ 140 individual cells of at least 4 independent biological samples. Statistics: One-way ANOVA with Tukey’s post hoc test. ns: no significant difference; *** p<0.001. (TIF) [file pgen.1007846.s002.tif]

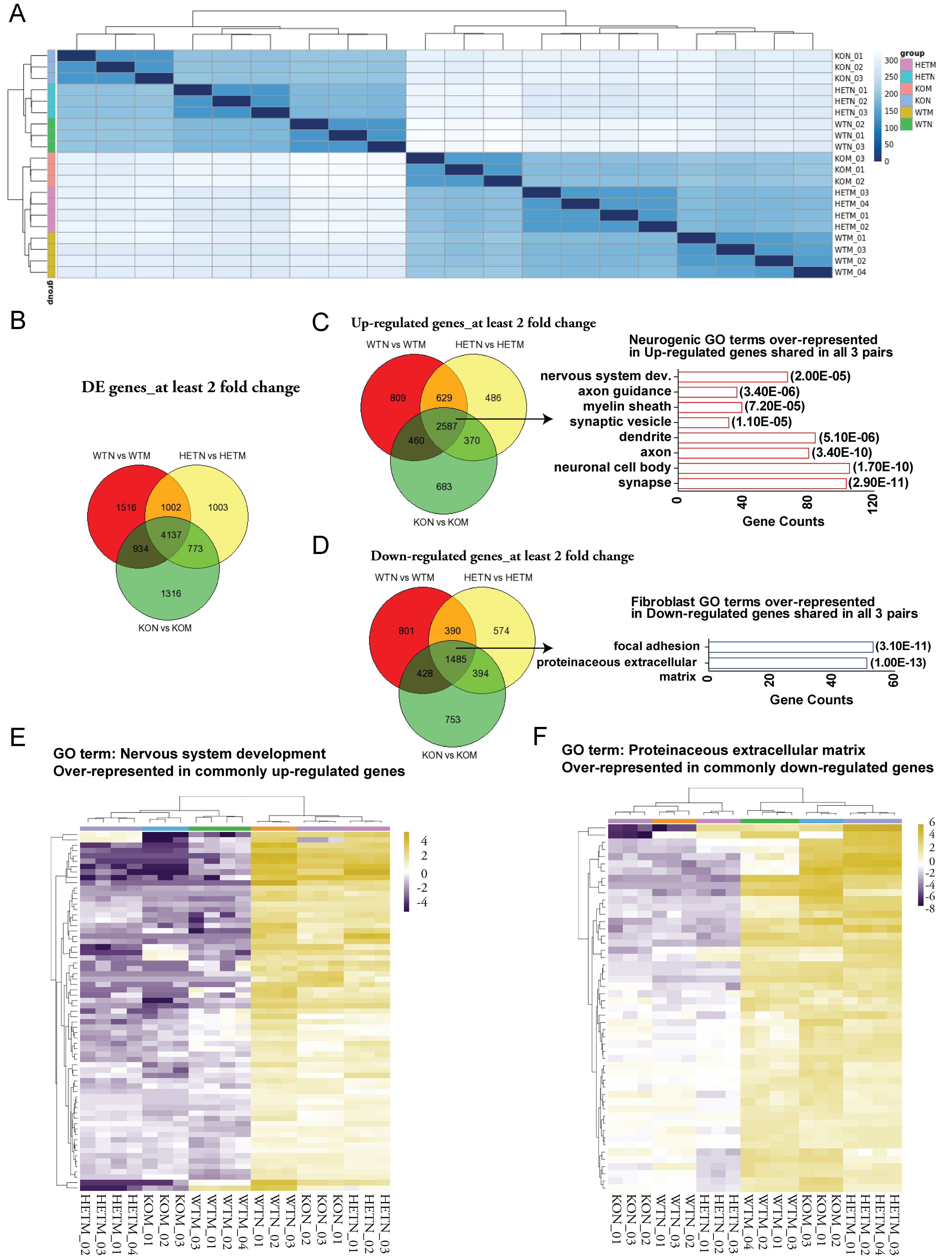

Supplement: S3 Fig — (A) Quantification of the similarity in the transcriptomes of transcriptomes of MEFs (WTM, HETM and KOM) and CiNeurons (WTN, HETN and KON). Euclidean distances were calculated from regularized log-transformed read counts. MEFs are clustered away from the CiNeurons. (B) Venn diagram shows the genes differentially expressed by at least 2 fold in each CiNeuron when compared to the MEF counterpart. The majority of DE genes are shared by three groups. (C) Venn diagram shows the genes up-regulated by at least 2 fold in each CiNeuron in comparison to the MEF counterpart. The commonly up-regulated genes shared by 3 genetic backgrounds were subject to GO enrichment analysis. The significantly over-represented terms related to neuron are shown (gene counts of the GO term ≥ 30, fold of enrichment ≥ 1.5 and p value of enrichment <0.01). (D) The same analysis is performed with the commonly down-regulated genes and the significantly enriched, fibroblast-related GO terms: focal adhesion and extracellular matrix are shown. (E) Heatmap showing the expression level of genes associated with GO term: Nervous system development that are commonly up-regulated in all CiNeurons. (F) Heatmap showing the expression level of genes associated with GO term: Proteinaceous extracellular matrix that are commonly down-regulated in all CiNeurons. Scale bar: Log2 CPM. (TIF) [file pgen.1007846.s003.tif]

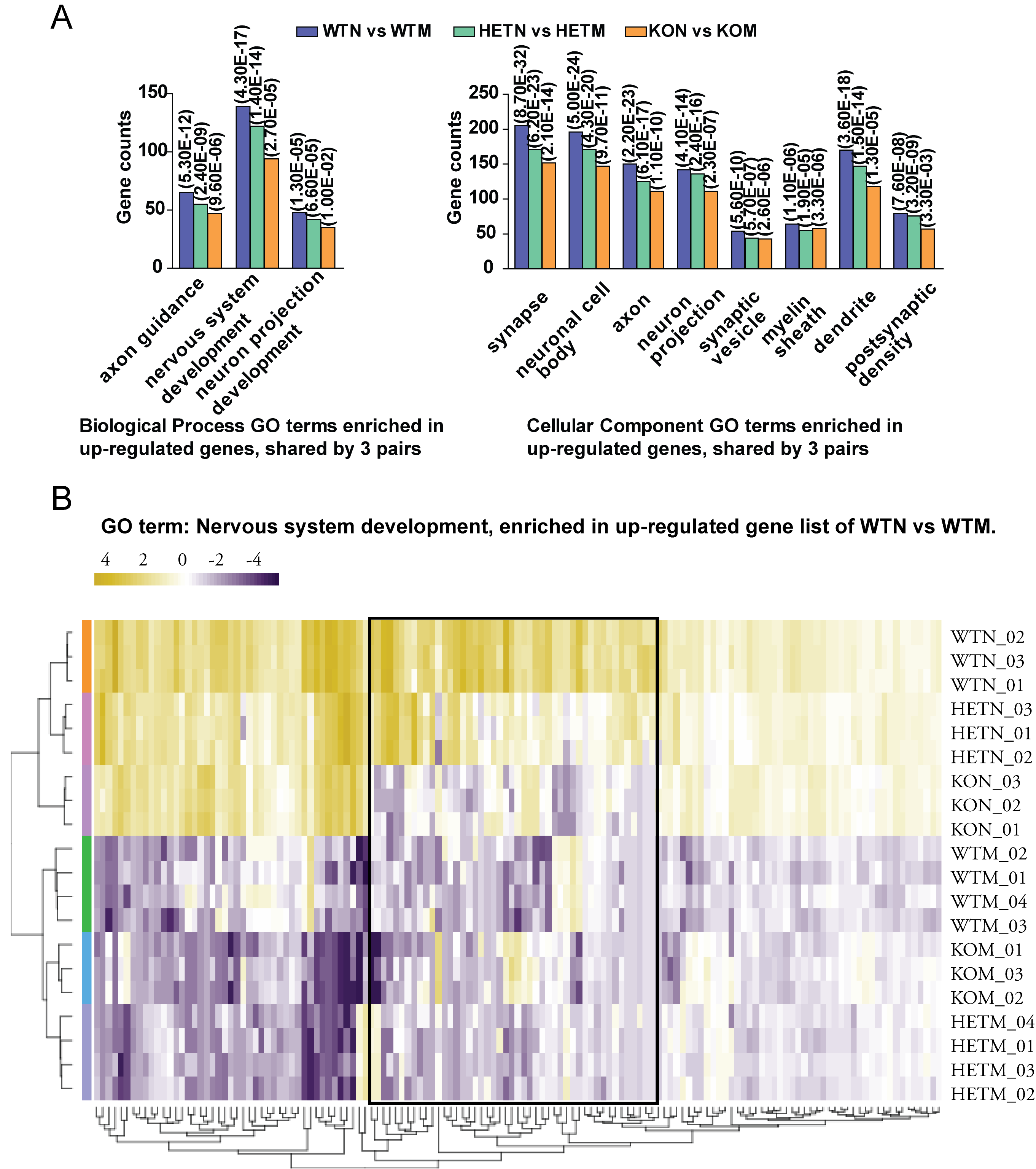

Supplement: S4 Fig — (A) Genes up-regulated in CiNeuron of each genetic background are subject to GO enrichment analysis respectively. The significantly enriched, neuron-related GO terms in Biological Processes Cellular Components are shown. Y-axis shows the number of gene counts in each GO term. Numbers in parentheses show the p value of enrichment of each GO term. (B) Genes that are associated with nervous system development and are significantly up-regulated in WTN vs WTM are selected. Heatmap shows the expression level of those genes in all MEFs and CiNeurons. Black frame highlights the genes that failed to be up-regulated in CiNeuron of KO background. Scale bar: log2 CPM. (TIF) [file pgen.1007846.s004.tif]

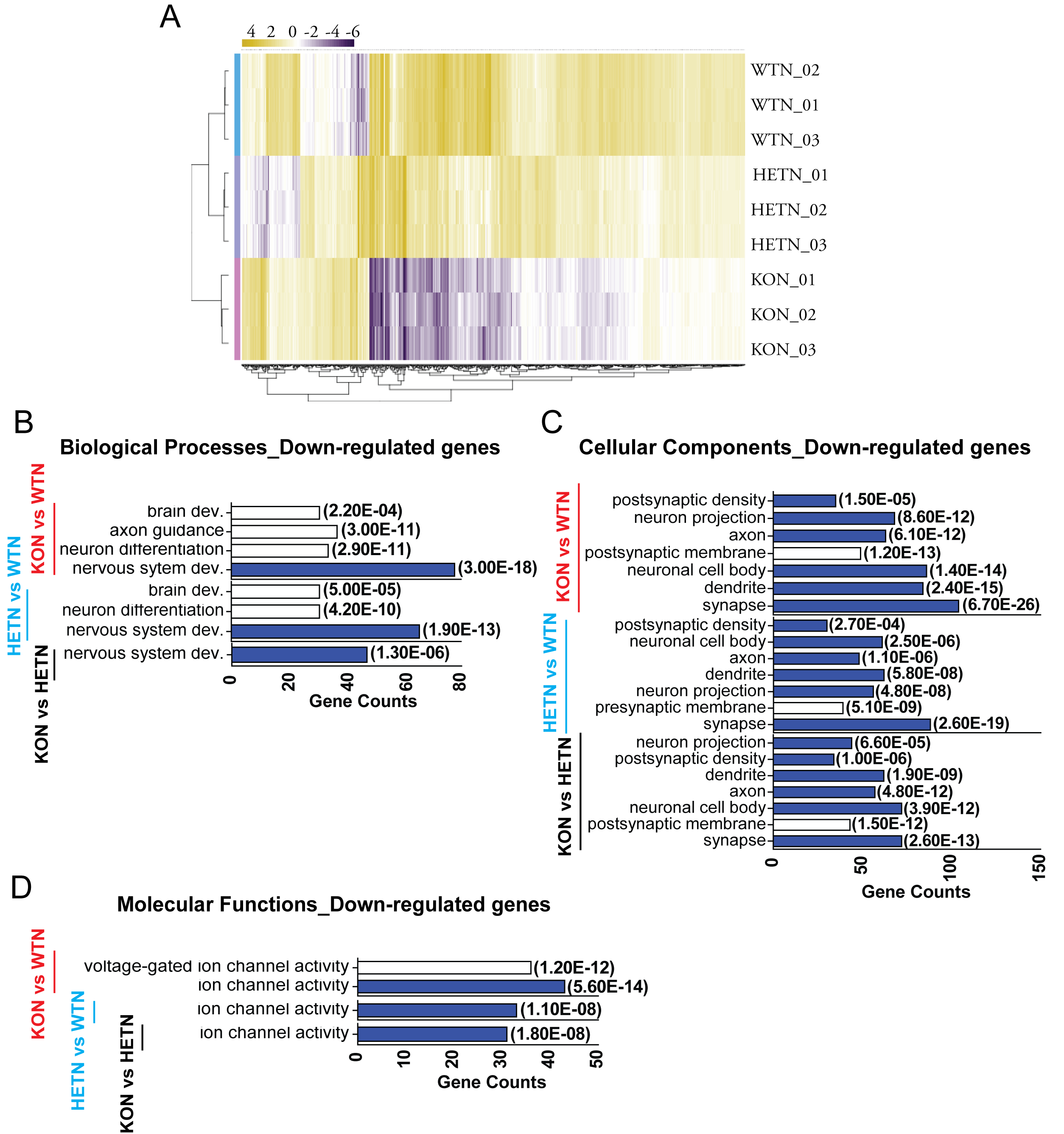

Supplement: S5 Fig — (A) Genes are selected when they are differentially expressed by at least 2 fold in WTN vs KON comparison, and are also significantly changed in HETN vs WTN and KON vs HETN comparisons. Clustering is based on the CV of gene expression. Scale bar: log2 CPM. (B-D) Genes down-regulated in each comparison group (less β-actin background compared to more β-actin background, e.g. KON vs WTN) are subject to GO enrichment analysis. The significantly enriched, neuron-related GO terms are shown in (B) Biological processes, (C) Cellular Components and (D) Molecular functions. (TIF) [file pgen.1007846.s005.tif]

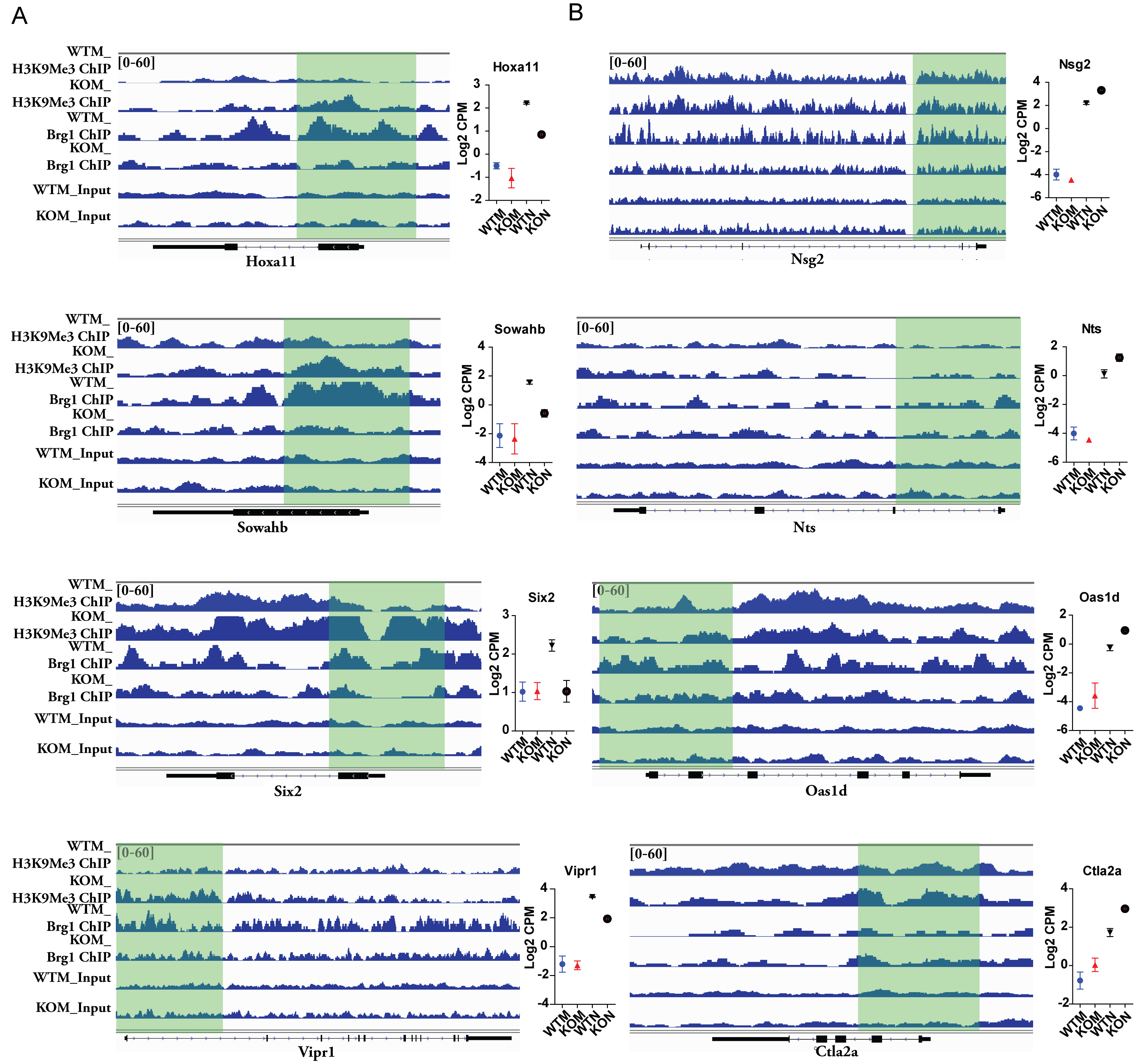

Supplement: S6 Fig — (A) Examples showing H3K9Me3 and Brg1 ChIP-seq data of MEFs at gene loci that are down-regulated in KON vs WTN. The y-axis data range represents RPKM (Reads Per Kilobase of sequence range per Million mapped reads) per bin. The y-axis of tracks in the same image were set as the same range. Gene body position (exon: box, intron: line) are shown below the tracks. The transcription start site (TSS) of each gene is highlighted. The plots are the summary of relative gene expression level of at least 3 biological replicates from RNA-seq data. (B) Examples showing H3K9Me3 and Brg1 ChIP-seq data of MEFs at gene loci that are up-regulated in KON vs WTN. (TIF) [file pgen.1007846.s006.tif]

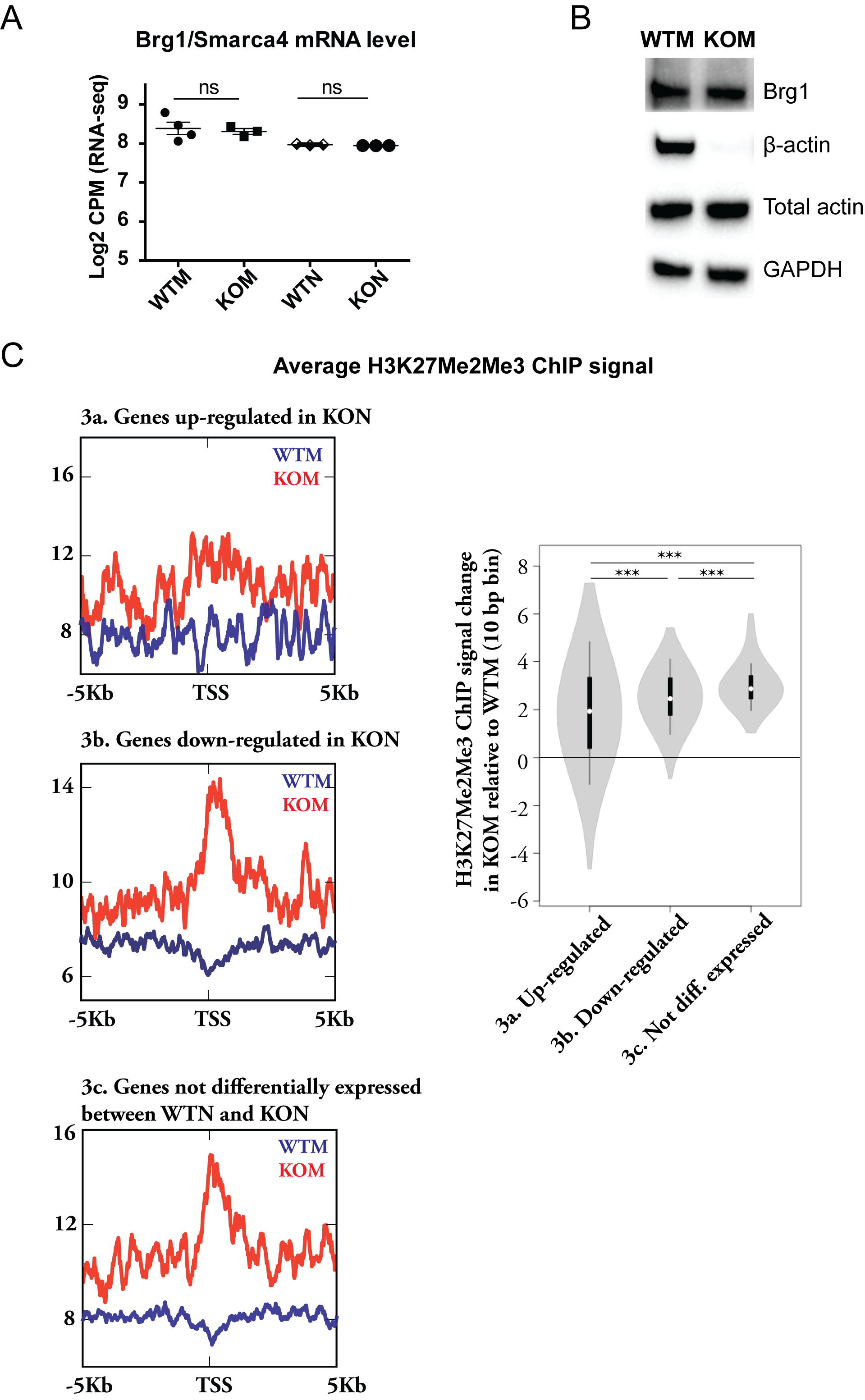

Supplement: S7 Fig — (A) Brg1/Smarca4 mRNA level in MEFs and Neurons by RNA-seq analysis. Each dot in the plot is a biological replicate. Student’s t-test, ns: no significant difference. (B) Western blot analysis of the Brg1 protein level in whole cell lysate of WT and KO MEFs. GAPDH is used as loading control. The Brg1 and total actin protein levels are comparable between WTM and KOM cells. (C) Left panel: Average H3K27Me2Me3 Chip-seq signal (RPKM) within ± 5kb of TSS of genes in gene lists of 3a, 3b, and 3c of Fig 6A between WTM and KOM. Right panel: The difference of H3K27Me2Me3 Chip-seq signal in each10 bp bin within ± 5kb of TSS between KOM and WTM was calculated. Violin diagram shows the distribution of the relative ChIP signal difference at each 10 bp bin between KOM and WTM. One-way ANOVA with Tukey’s post hoc test: *** p<0.001. (TIF) [file pgen.1007846.s007.tif]

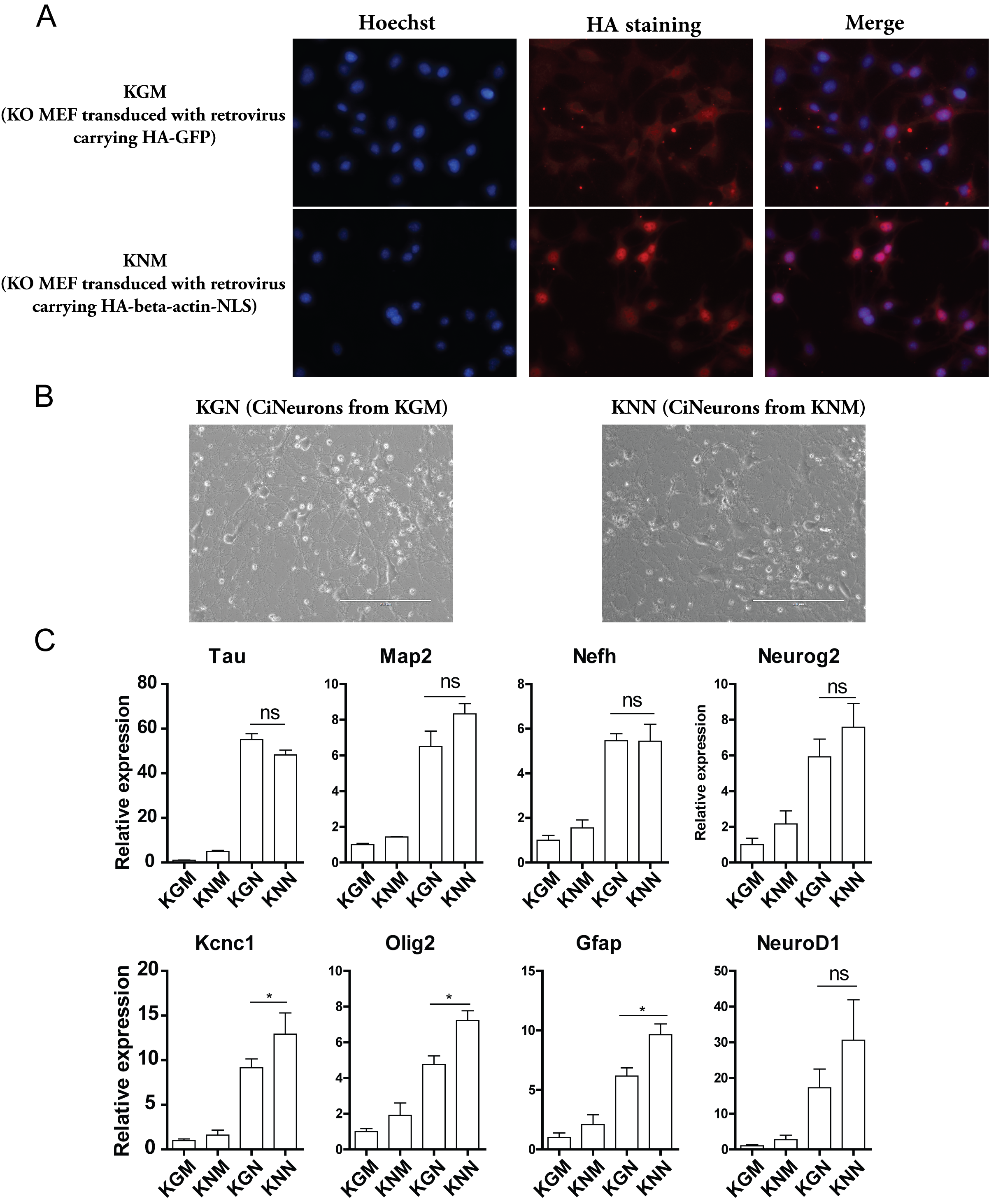

Supplement: S8 Fig — (A) Immunofluorescence staining of KOM cells with re-introduced HA-β-actin-NLS (KNM cells). The HA-GFP is used as a control for retroviral transduction (KGM cells). The HA-β-actin-NLS staining is preferentially enriched in the cellular nucleus. (B) Morphology of the CiNeurons (KGN & KNN) induced from KGM and KNM cells. (C) qPCR analysis of the relative expression level of neuron markers among KGM, KNM, KGN, KNN cells. The gene expression difference between induced neurons KGN and KNN are analyzed by Student’s t-test, ns: no significant difference; * p<0.05. Results are pooled data of 3 independent biological samples. (TIF) [file pgen.1007846.s008.tif]
