# Supplementary material for: β-actin regulates a heterochromatin landscape essential for optimal induction of neuronal programs during direct reprograming
Source: PLoS Genet. 2018 Dec 17;14(12):e1007846. doi: 10.1371/journal.pgen.1007846 (PMC6312353; doi:10.1371/journal.pgen.1007846)
Supplement: S1 Table — Genes commonly up-regulated or down-regulated in all CiNeurons when compared to the MEF counterpart are selected respectively, and are subject to GO enrichment analysis. The significantly over-represented GO terms in Biological Process, Cellular Component and Molecular function are shown, and their gene counts enrichment p value, and fold of enrichment. (Criteria for GO terms to be considered as significantly over-represented: Gene count at least 30, P value <0.01, Fold of enrichment at least 1.5). (DOCX) [file pgen.1007846.s009.docx]

**S1 Table.** GO enrichment analysis of commonly up-regulated or down-regulated genes shared by CiNeurons in comparison to the MEF counterpart.

| **GO terms enriched in commonly up-regulated genes**  **(up-regulated by at least 2 fold in all pairs of comparisons: CiNeurons relative to MEF counterparts, yellow indicates neuron-related GO terms)** | | | |
| --- | --- | --- | --- |
| **Biological process** | | | |
| GO term | Gene Count | P value | Fold of enrichment |
| ion transport | 115 | 2.80E-11 | 1.9 |
| transmembrane transport | 75 | 1.50E-08 | 2 |
| locomotory behavior | 33 | 3.20E-08 | 2.9 |
| axon guidance | 36 | 3.40E-06 | 2.3 |
| nervous system development | 67 | 2.00E-05 | 1.7 |
| cell adhesion | 81 | 2.60E-05 | 1.6 |
| negative regulation of cell proliferation | 67 | 3.60E-05 | 1.7 |
| negative regulation of neuron apoptotic process | 35 | 4.50E-05 | 2.1 |
| calcium ion transport | 30 | 2.80E-04 | 2 |
| immune system process | 63 | 3.40E-04 | 1.6 |
| positive regulation of protein phosphorylation | 35 | 7.20E-04 | 1.8 |
| homophilic cell adhesion via plasma membrane adhesion molecules | 32 | 7.40E-04 | 1.9 |
| lipid metabolic process | 71 | 8.20E-04 | 1.5 |
| translation | 63 | 1.10E-03 | 1.5 |
| carbohydrate metabolic process | 37 | 1.50E-03 | 1.7 |
| brain development | 38 | 1.80E-03 | 1.7 |
| response to hypoxia | 34 | 3.00E-03 | 1.7 |
| response to drug | 53 | 3.20E-03 | 1.5 |
| angiogenesis | 40 | 3.40E-03 | 1.6 |
| cell surface receptor signaling pathway | 37 | 4.20E-03 | 1.6 |
| response to lipopolysaccharide | 34 | 4.50E-03 | 1.6 |
| positive regulation of ERK1 and ERK2 cascade | 30 | 2.20E-02 | 1.5 |
| **Cellular component** | | | |
| GO term | Gene Count | P value | Fold of enrichment |
| ribosome | 58 | 3.80E-14 | 3 |
| lysosome | 80 | 9.60E-13 | 2.3 |
| synapse | 103 | 2.90E-11 | 2 |
| neuronal cell body | 105 | 1.70E-10 | 1.9 |
| axon | 80 | 3.40E-10 | 2.1 |
| lysosomal membrane | 50 | 1.30E-06 | 2.1 |
| cell surface | 104 | 1.90E-06 | 1.6 |
| neuron projection | 76 | 2.00E-06 | 1.7 |
| dendrite | 84 | 5.10E-06 | 1.6 |
| synaptic vesicle | 31 | 1.10E-05 | 2.4 |
| cell junction | 112 | 1.20E-05 | 1.5 |
| endosome | 88 | 2.90E-05 | 1.6 |
| myelin sheath | 39 | 7.20E-05 | 2 |
| membrane raft | 48 | 1.40E-04 | 1.8 |
| postsynaptic density | 43 | 4.80E-04 | 1.7 |
| intracellular ribonucleoprotein complex | 52 | 1.30E-03 | 1.6 |
| mitochondrial inner membrane | 60 | 1.70E-03 | 1.5 |
| transcription factor complex | 43 | 4.20E-03 | 1.5 |
| **Molecular function** | | | |
| GO term | Gene Count | P value | Fold of enrichment |
| structural constituent of ribosome | 63 | 8.50E-10 | 2.3 |
| protein heterodimerization activity | 86 | 1.60E-05 | 1.6 |
| calcium ion binding | 107 | 6.80E-05 | 1.5 |
| calmodulin binding | 37 | 1.40E-04 | 1.9 |
| receptor binding | 67 | 3.50E-04 | 1.5 |
| transcriptional activator activity, RNA polymerase II core promoter proximal region sequence-specific binding | 47 | 7.00E-04 | 1.7 |
| ion channel activity | 31 | 3.20E-03 | 1.7 |

| **GO terms enriched in commonly down-regulated genes**  **(down-regulated by at least 2 fold in all pairs of comparisons: CiNeurons relative to MEF counterparts, yellow indicates fibroblast-specific GO term)** | | | |
| --- | --- | --- | --- |
| **Biological process** | | | |
| GO term | Gene Count | P value | Fold of enrichment |
| cell cycle | 87 | 8.60E-19 | 2.9 |
| mitotic nuclear division | 52 | 3.20E-16 | 3.8 |
| cell division | 60 | 1.90E-15 | 3.2 |
| cell adhesion | 46 | 3.80E-05 | 1.9 |
| heart development | 30 | 4.00E-05 | 2.3 |
| negative regulation of transcription from RNA polymerase II promoter | 59 | 2.40E-04 | 1.6 |
| positive regulation of cell proliferation | 45 | 8.80E-04 | 1.7 |
| positive regulation of transcription from RNA polymerase II promoter | 72 | 1.20E-03 | 1.5 |
| cellular response to DNA damage stimulus | 36 | 1.80E-03 | 1.7 |
| positive regulation of gene expression | 32 | 8.80E-03 | 1.6 |
| **Cellular component** | | | |
| GO term | Gene Count | P value | Fold of enrichment |
| proteinaceous extracellular matrix | 51 | 1.00E-13 | 3.3 |
| cytoskeleton | 112 | 1.50E-13 | 2.1 |
| chromosome, centromeric region | 30 | 2.60E-11 | 4.4 |
| focal adhesion | 53 | 3.10E-11 | 2.8 |
| extracellular matrix | 43 | 2.70E-10 | 3 |
| chromosome | 46 | 1.20E-09 | 2.8 |
| nucleoplasm | 143 | 1.90E-07 | 1.5 |
| cell projection | 65 | 1.20E-06 | 1.9 |
| nucleolus | 68 | 3.90E-05 | 1.7 |
| centrosome | 40 | 1.50E-04 | 1.9 |
| perinuclear region of cytoplasm | 54 | 6.20E-04 | 1.6 |
| **Molecular function** | | | |
| GO term | Gene Count | P value | Fold of enrichment |
| poly(A) RNA binding | 94 | 3.40E-07 | 1.7 |
| zinc ion binding | 88 | 3.10E-06 | 1.7 |
| actin binding | 38 | 4.90E-06 | 2.3 |
| chromatin binding | 47 | 6.10E-06 | 2 |
| RNA binding | 62 | 2.50E-04 | 1.6 |
