# Supplementary material for: β-actin regulates a heterochromatin landscape essential for optimal induction of neuronal programs during direct reprograming
Source: PLoS Genet. 2018 Dec 17;14(12):e1007846. doi: 10.1371/journal.pgen.1007846 (PMC6312353; doi:10.1371/journal.pgen.1007846)
Supplement: S2 Table — Genes up-regulated in each comparison group of CiNeurons (less β-actin background compared to more β-actin background, e.g. KON vs HETN) are subject to GO enrichment analysis. The significantly over-represented GO terms in Biological Process, Cellular Component and Molecular Function are shown. (Genes up-regulated in A (A vs B) by at least 2 fold; Criteria for GO terms to be considered as significantly over-represented: Gene count at least 30, P value <0.01, Fold of enrichment at least 1.5). (DOCX) [file pgen.1007846.s010.docx]

**S2 Table.** GO enrichment analysis of genes up-regulated among CiNeurons.

| **Genes up-regulated in KON vs HETN** | | | |
| --- | --- | --- | --- |
| **Biological process** | | | |
| GO terms | Gene count | P value | Fold of Enrichment |
| cell adhesion | 49 | 4.80E-09 | 2.6 |
| lipid metabolic process | 33 | 1.40E-03 | 1.8 |
| ion transport | 39 | 1.80E-03 | 1.7 |
| oxidation-reduction process | 41 | 7.20E-03 | 1.5 |
| **Cellular component** | | | |
| GO terms | Gene count | P value | Fold of Enrichment |
| extracellular region | 152 | 1.30E-20 | 2.2 |
| proteinaceous extracellular matrix | 45 | 4.00E-13 | 3.6 |
| extracellular exosome | 177 | 2.90E-12 | 1.7 |
| extracellular space | 114 | 2.60E-11 | 1.9 |
| extracellular matrix | 37 | 2.10E-09 | 3.2 |
| cell surface | 47 | 5.40E-05 | 1.9 |
| integral component of plasma membrane | 72 | 7.80E-05 | 1.6 |
| cell junction | 49 | 3.10E-04 | 1.7 |
| cytoplasmic vesicle | 41 | 3.80E-03 | 1.6 |
| **Molecular function** | | | |
| GO terms | Gene count | P value | Fold of Enrichment |
| calcium ion binding | 71 | 1.10E-12 | 2.5 |
| oxidoreductase activity | 42 | 6.80E-04 | 1.7 |
| receptor binding | 31 | 1.20E-03 | 1.9 |

| **Genes up-regulated in KON vs WTN** | | | |
| --- | --- | --- | --- |
| **Biological process** | | | |
| No GO terms found to be significantly over-represented | | | |
| **Cellular component** | | | |
| GO terms | Gene count | P value | Fold of Enrichment |
| extracellular region | 64 | 1.30E-06 | 1.9 |
| extracellular space | 49 | 4.60E-04 | 1.7 |
| extracellular exosome | 76 | 5.90E-04 | 1.5 |
| integral component of plasma membrane | 39 | 6.70E-04 | 1.8 |
| **Molecular function** | | | |
| GO terms | Gene count | P value | Fold of Enrichment |
| calcium ion binding | 39 | 2.30E-08 | 2.8 |

| **Genes up-regulated in HETN vs WTN** | | | |
| --- | --- | --- | --- |
| **Biological process** | | | |
| GO terms | Gene count | P value | Fold of Enrichment |
| cell cycle | 49 | 9.80E-09 | 2.5 |
| mitotic nuclear division | 30 | 2.00E-08 | 3.4 |
| cell division | 35 | 4.30E-08 | 2.9 |
| cellular response to DNA damage stimulus | 34 | 1.90E-06 | 2.5 |
| cell adhesion | 30 | 9.20E-04 | 1.9 |
| multicellular organism development | 48 | 8.70E-03 | 1.5 |
| **Cellular component** | | | |
| GO terms | Gene count | P value | Fold of Enrichment |
| nucleus | 240 | 8.00E-06 | 1.3 |
| cytoskeleton | 59 | 1.10E-04 | 1.7 |
| **Molecular function** | | | |
| GO terms | Gene count | P value | Fold of Enrichment |
| calcium ion binding | 45 | 1.20E-05 | 2 |
| DNA binding | 92 | 1.20E-05 | 1.6 |
| transcription factor activity, sequence-specific DNA binding | 48 | 3.60E-04 | 1.7 |
| sequence-specific DNA binding | 36 | 1.10E-03 | 1.8 |
