# Supplementary material for: β-actin regulates a heterochromatin landscape essential for optimal induction of neuronal programs during direct reprograming
Source: PLoS Genet. 2018 Dec 17;14(12):e1007846. doi: 10.1371/journal.pgen.1007846 (PMC6312353; doi:10.1371/journal.pgen.1007846)
Supplement: S3 Table — The final lists of filter genes 3a, 3b and 3c in Fig 7A are subject to GO enrichment analysis. The significantly over-represented GO terms in Biological Process, Cellular Component and Molecular Function are shown. (Criteria for GO terms to be considered as significantly over-represented: P value <0.01, Fold of enrichment at least 1.5). (DOCX) [file pgen.1007846.s011.docx]

**S3 Table.** GO enrichment analysis of gene lists filtered in Figure 7A.

| **3a. genes Up-regulated in KONvsWTN** | | | |
| --- | --- | --- | --- |
| **Biological process** | | | |
| GO Term | Gene Count | P value | Fold of Enrichment |
| regulation of energy homeostasis | 3 | 2.10E-03 | 43.1 |
| cell adhesion | 8 | 6.90E-03 | 3.6 |
| **Cellular component** | | | |
| GO Term | Gene Count | P value | Fold of Enrichment |
| extracellular region | 17 | 6.30E-03 | 2.1 |
| extracellular space | 15 | 9.10E-03 | 2.1 |
| extracellular exosome | 22 | 9.50E-03 | 1.8 |
| **Molecular function** | | | |
| No GO terms to be significantly over-represented | | | |

| **3b. genes down-regulated in KONvsWTN** | | | |
| --- | --- | --- | --- |
| **Biological process** | | | |
| GO Term | Gene Count | P value | Fold of Enrichment |
| nervous system development | 47 | 7.30E-16 | 4.1 |
| ion transport | 56 | 8.20E-14 | 3.2 |
| regulation of ion transmembrane transport | 26 | 3.20E-13 | 6.3 |
| multicellular organism development | 75 | 3.50E-12 | 2.4 |
| potassium ion transmembrane transport | 20 | 2.10E-11 | 7.3 |
| potassium ion transport | 23 | 3.40E-11 | 6 |
| axon guidance | 23 | 8.40E-10 | 5.1 |
| transmembrane transport | 34 | 2.30E-08 | 3.1 |
| memory | 16 | 2.60E-08 | 6.3 |
| regulation of membrane potential | 16 | 2.20E-07 | 5.4 |
| hippocampus development | 13 | 1.90E-06 | 5.9 |
| positive regulation of transcription, DNA-templated | 40 | 2.70E-06 | 2.3 |
| cAMP-mediated signaling | 9 | 4.90E-06 | 9 |
| chemical synaptic transmission | 19 | 5.20E-06 | 3.6 |
| calcium ion transport | 17 | 6.10E-06 | 4 |
| signal transduction | 66 | 1.60E-05 | 1.7 |
| transport | 87 | 2.20E-05 | 1.6 |
| glial cell differentiation | 7 | 2.80E-05 | 11 |
| neuron differentiation | 15 | 3.20E-05 | 3.9 |
| cellular calcium ion homeostasis | 13 | 5.00E-05 | 4.3 |
| synapse assembly | 9 | 5.50E-05 | 6.6 |
| pattern specification process | 10 | 5.80E-05 | 5.7 |
| adenylate cyclase-modulating G-protein coupled receptor signaling pathway | 8 | 6.00E-05 | 7.7 |
| cell-cell signaling | 13 | 6.70E-05 | 4.1 |
| neuropeptide signaling pathway | 11 | 9.70E-05 | 4.8 |
| regulation of short-term neuronal synaptic plasticity | 6 | 1.20E-04 | 11.6 |
| adenylate cyclase-activating G-protein coupled receptor signaling pathway | 9 | 1.20E-04 | 5.9 |
| cell differentiation | 44 | 1.20E-04 | 1.9 |
| learning | 10 | 1.60E-04 | 5 |
| patterning of blood vessels | 8 | 1.80E-04 | 6.6 |
| cell adhesion | 31 | 1.90E-04 | 2.1 |
| dopaminergic neuron differentiation | 7 | 2.00E-04 | 7.9 |
| positive regulation of synapse assembly | 10 | 2.60E-04 | 4.7 |
| inner ear morphogenesis | 10 | 3.20E-04 | 4.6 |
| positive regulation of transcription from RNA polymerase II promoter | 51 | 3.30E-04 | 1.7 |
| positive regulation of heart rate | 6 | 3.40E-04 | 9.4 |
| neuron migration | 13 | 4.00E-04 | 3.4 |
| feeding behavior | 7 | 5.00E-04 | 6.8 |
| heart looping | 9 | 5.50E-04 | 4.8 |
| cell chemotaxis | 10 | 5.80E-04 | 4.2 |
| odontogenesis of dentin-containing tooth | 9 | 6.10E-04 | 4.7 |
| regulation of calcium ion transport | 7 | 8.00E-04 | 6.2 |
| neuron fate specification | 5 | 8.80E-04 | 11 |
| sensory perception of sound | 13 | 1.00E-03 | 3.1 |
| negative regulation of canonical Wnt signaling pathway | 11 | 1.10E-03 | 3.5 |
| regulation of transcription from RNA polymerase II promoter | 25 | 1.10E-03 | 2.1 |
| calcium ion transmembrane transport | 10 | 1.40E-03 | 3.7 |
| homophilic cell adhesion via plasma membrane adhesion molecules | 14 | 1.40E-03 | 2.8 |
| Notch signaling pathway | 12 | 1.50E-03 | 3.2 |
| central nervous system development | 10 | 1.50E-03 | 3.7 |
| skin development | 8 | 1.80E-03 | 4.5 |
| neuron development | 8 | 1.80E-03 | 4.5 |
| negative regulation of neuron differentiation | 9 | 2.10E-03 | 3.9 |
| anterior/posterior pattern specification | 11 | 2.20E-03 | 3.2 |
| sensory perception of pain | 9 | 2.30E-03 | 3.8 |
| male genitalia development | 5 | 2.80E-03 | 8.2 |
| protein homooligomerization | 15 | 2.80E-03 | 2.5 |
| response to lipopolysaccharide | 15 | 2.80E-03 | 2.5 |
| ion transmembrane transport | 7 | 2.80E-03 | 4.9 |
| cellular response to organic substance | 6 | 3.00E-03 | 6 |
| response to corticosterone | 5 | 3.30E-03 | 7.8 |
| negative regulation of cell proliferation | 23 | 3.50E-03 | 2 |
| long-term synaptic potentiation | 7 | 3.50E-03 | 4.7 |
| neural crest cell migration | 7 | 3.50E-03 | 4.7 |
| negative regulation of neuron apoptotic process | 13 | 3.60E-03 | 2.7 |
| cerebellum development | 7 | 3.90E-03 | 4.6 |
| palate development | 9 | 4.30E-03 | 3.5 |
| regulation of insulin secretion involved in cellular response to glucose stimulus | 5 | 4.70E-03 | 7.1 |
| retina layer formation | 5 | 4.70E-03 | 7.1 |
| associative learning | 6 | 4.90E-03 | 5.3 |
| embryonic pattern specification | 5 | 5.50E-03 | 6.8 |
| adult behavior | 6 | 5.60E-03 | 5.2 |
| thymus development | 7 | 6.30E-03 | 4.2 |
| kidney development | 11 | 6.30E-03 | 2.8 |
| positive regulation of apoptotic process | 20 | 7.00E-03 | 2 |
| potassium ion import | 5 | 7.40E-03 | 6.3 |
| response to morphine | 5 | 7.40E-03 | 6.3 |
| response to drug | 20 | 7.90E-03 | 1.9 |
| neuromuscular process controlling balance | 7 | 8.10E-03 | 4 |
| response to immobilization stress | 5 | 8.50E-03 | 6.1 |
| response to ethanol | 10 | 9.30E-03 | 2.8 |
| ephrin receptor signaling pathway | 6 | 9.40E-03 | 4.6 |
| calcium ion-regulated exocytosis of neurotransmitter | 5 | 9.60E-03 | 5.9 |
| regulation of long-term neuronal synaptic plasticity | 5 | 9.60E-03 | 5.9 |
| **Cellular component** | | | |
| GO Term | Gene Count | P value | Fold of Enrichment |
| synapse | 66 | 1.90E-23 | 4.3 |
| membrane | 312 | 6.60E-18 | 1.5 |
| neuronal cell body | 59 | 1.70E-17 | 3.7 |
| dendrite | 56 | 2.80E-17 | 3.8 |
| cell junction | 69 | 3.30E-17 | 3.2 |
| axon | 46 | 1.60E-15 | 4.1 |
| plasma membrane | 225 | 7.90E-13 | 1.5 |
| integral component of plasma membrane | 79 | 4.70E-12 | 2.3 |
| postsynaptic membrane | 31 | 6.20E-12 | 4.6 |
| neuron projection | 43 | 1.10E-11 | 3.4 |
| terminal bouton | 17 | 2.80E-07 | 5 |
| presynaptic membrane | 14 | 3.40E-07 | 6.2 |
| voltage-gated potassium channel complex | 14 | 6.40E-07 | 5.9 |
| membrane raft | 23 | 1.60E-05 | 2.9 |
| synaptic vesicle | 15 | 2.70E-05 | 3.9 |
| perikaryon | 16 | 5.00E-05 | 3.5 |
| axon terminus | 12 | 5.40E-05 | 4.6 |
| dendrite membrane | 7 | 1.00E-04 | 8.9 |
| cone cell pedicle | 4 | 1.10E-04 | 33.1 |
| postsynaptic density | 20 | 1.20E-04 | 2.8 |
| dendritic spine | 15 | 1.60E-04 | 3.4 |
| neuronal cell body membrane | 7 | 1.90E-04 | 8 |
| extracellular region | 79 | 3.20E-04 | 1.5 |
| synaptic vesicle membrane | 9 | 4.70E-04 | 4.9 |
| basolateral plasma membrane | 16 | 1.30E-03 | 2.6 |
| GABA-A receptor complex | 5 | 1.80E-03 | 9.2 |
| presynaptic active zone | 6 | 1.90E-03 | 6.6 |
| dendritic shaft | 8 | 2.30E-03 | 4.3 |
| chloride channel complex | 7 | 2.50E-03 | 5 |
| sarcolemma | 11 | 3.00E-03 | 3.1 |
| inhibitory synapse | 5 | 3.30E-03 | 7.9 |
| anchored component of membrane | 12 | 3.70E-03 | 2.8 |
| voltage-gated calcium channel complex | 5 | 5.40E-03 | 6.9 |
| growth cone | 12 | 8.90E-03 | 2.5 |
| **Molecular function** | | | |
| GO Term | Gene Count | P value | Fold of Enrichment |
| ion channel activity | 29 | 2.80E-13 | 5.6 |
| voltage-gated ion channel activity | 26 | 2.90E-13 | 6.4 |
| sequence-specific DNA binding | 57 | 7.30E-13 | 2.9 |
| potassium channel activity | 19 | 2.40E-11 | 7.8 |
| voltage-gated potassium channel activity | 17 | 2.40E-10 | 7.9 |
| delayed rectifier potassium channel activity | 10 | 3.20E-07 | 10.2 |
| transcriptional activator activity, RNA polymerase II core promoter proximal region sequence-specific binding | 26 | 8.70E-07 | 3.2 |
| calcium ion binding | 45 | 4.50E-06 | 2.1 |
| cAMP binding | 8 | 9.40E-06 | 10.1 |
| RNA polymerase II core promoter proximal region sequence-specific DNA binding | 28 | 1.60E-05 | 2.6 |
| transcription factor activity, sequence-specific DNA binding | 48 | 1.50E-04 | 1.8 |
| RNA polymerase II regulatory region sequence-specific DNA binding | 19 | 1.50E-04 | 2.8 |
| extracellular ligand-gated ion channel activity | 8 | 2.20E-04 | 6.4 |
| PDZ domain binding | 13 | 2.20E-04 | 3.7 |
| [heparan sulfate]-glucosamine 3-sulfotransferase 1 activity | 4 | 2.70E-04 | 26.2 |
| signal transducer activity | 36 | 8.00E-04 | 1.8 |
| calcium channel activity | 10 | 1.20E-03 | 3.8 |
| calmodulin binding | 15 | 1.40E-03 | 2.7 |
| transcription regulatory region DNA binding | 17 | 2.20E-03 | 2.4 |
| GABA-A receptor activity | 5 | 2.30E-03 | 8.6 |
| phosphatidylinositol-4,5-bisphosphate binding | 8 | 2.50E-03 | 4.3 |
| ion channel binding | 11 | 3.70E-03 | 3 |
| protein heterodimerization activity | 28 | 4.50E-03 | 1.8 |
| transcriptional activator activity, RNA polymerase II transcription regulatory region sequence-specific binding | 10 | 4.50E-03 | 3.1 |
| transmembrane receptor protein tyrosine kinase activity | 7 | 5.80E-03 | 4.2 |
| transcriptional repressor activity, RNA polymerase II core promoter proximal region sequence-specific binding | 11 | 6.20E-03 | 2.8 |
| ephrin receptor activity | 4 | 8.00E-03 | 9.4 |
| Toll-like receptor 4 binding | 3 | 8.70E-03 | 19.6 |

| **3c. genes_no change in KONvsWTN** | | | |
| --- | --- | --- | --- |
| **Biological process** | | | |
| GO Term | Gene Count | P value | Fold of Enrichment |
| nucleosome assembly | 18 | 8.80E-06 | 3.6 |
| cation transport | 13 | 1.70E-04 | 3.7 |
| neurotransmitter secretion | 8 | 2.80E-04 | 5.9 |
| transmembrane transport | 34 | 3.60E-04 | 1.9 |
| ion transport | 48 | 3.80E-04 | 1.7 |
| calcium ion transport | 18 | 4.30E-04 | 2.7 |
| positive regulation of gene expression, epigenetic | 8 | 8.20E-04 | 5 |
| cellular calcium ion homeostasis | 14 | 9.80E-04 | 2.9 |
| potassium ion transmembrane transport | 13 | 1.20E-03 | 3 |
| ion transmembrane transport | 9 | 1.60E-03 | 4 |
| positive regulation of dendrite extension | 6 | 1.70E-03 | 6.6 |
| homophilic cell adhesion via plasma membrane adhesion molecules | 18 | 2.20E-03 | 2.3 |
| DNA methylation on cytosine | 7 | 3.70E-03 | 4.6 |
| social behavior | 9 | 4.00E-03 | 3.5 |
| DNA replication-dependent nucleosome assembly | 7 | 4.40E-03 | 4.4 |
| detection of temperature stimulus involved in sensory perception of pain | 5 | 4.70E-03 | 6.9 |
| locomotory behavior | 13 | 5.60E-03 | 2.5 |
| protein heterotetramerization | 8 | 6.70E-03 | 3.5 |
| negative regulation of megakaryocyte differentiation | 5 | 7.60E-03 | 6.1 |
| calcium ion transmembrane transport | 11 | 9.40E-03 | 2.6 |
| **Cellular component** | | | |
| GO Term | Gene Count | P value | Fold of Enrichment |
| nucleosome | 16 | 1.90E-04 | 3.1 |
| nuclear nucleosome | 10 | 2.00E-04 | 4.7 |
| presynaptic membrane | 12 | 8.50E-04 | 3.3 |
| neuronal cell body | 40 | 6.10E-03 | 1.6 |
| **Molecular function** | | | |
| GO Term | Gene Count | P value | Fold of Enrichment |
| calcium ion binding | 58 | 4.90E-05 | 1.7 |
| cation channel activity | 8 | 1.60E-03 | 4.5 |
| calcium channel activity | 12 | 2.50E-03 | 2.9 |
| metalloendopeptidase inhibitor activity | 5 | 2.60E-03 | 8.1 |
